# Supplementary material for: Challenges of early renal cancer detection: symptom patterns and incidental diagnosis rate in a multicentre prospective UK cohort of patients presenting with suspected renal cancer
Source: BMJ Open. 2020 May 11;10(5):e035938. doi: 10.1136/bmjopen-2019-035938 (PMC7223292; doi:10.1136/bmjopen-2019-035938)
Supplement: Supplementary data [file bmjopen-2019-035938supp001.pdf]

Supplementary Table 1. Study recruitment by centre

Centre ID	Total patients recruited n (% of total)	Recruitment period (months)*	Recruitment rate (pt/mo)
50	255 (36.1)	35	7.3
69	103 (14.6)	19	5.4
221	75 (10.6)	28	2.7
361	72 (10.3)	23	3.1
15	62 (8.8)	21	2.9
39	44 (6.2)	25	1.8
153	33 (4.7)	15	2.2
352	27 (3.8)	25	1.1
537	13 (1.8)	12	1.1
132	13 (1.8)	15	0.9
131	9 (1.3)	11	0.8

First patient registered July 2011; Last patient registered June 2014
* recruitment times vary based on the fact that centres opened to recruitment at different times
