# Supplementary material for: Challenges of early renal cancer detection: symptom patterns and incidental diagnosis rate in a multicentre prospective UK cohort of patients presenting with suspected renal cancer
Source: BMJ Open. 2020 May 11;10(5):e035938. doi: 10.1136/bmjopen-2019-035938 (PMC7223292; doi:10.1136/bmjopen-2019-035938)
Supplement: Supplementary data [file bmjopen-2019-035938supp002.pdf]

Supplementary Table 2. Comparison of current study patient characteristics with other RCC populations

Characteristic	Current Cohort (n=608)	UK RCC population data	US RCC population data n=104,000 Saad et al. 2019 [d]	Global RCC Study n=4288 (69.2% European cases) Laguna et al. 2014[e]
Age (years) median (range)	63.5 (29-92)	69[a]	<65 (52%)<br>>65 (48%)	62 (18-92)
Sex (M:F) (%)	65:35	63:37[b]	64:36	64:36
pT stage n (%)				
1	341 (57)	-	-	2954 (68)
2	78 (13)	-	-	553 (13)
3	172 (29)	-	-	617 (14)
4	4 (1)	-	-	32 (1)
Missing	1 (0)	-	-	195 (4)
TNM Stage n (%)				
I	341 (56)	4005 (43)[c]	68094 (65.1)	-
II	70 (12)	677 (7)	(Combined with I)	-
III	142 (23)	1560 (17)	16480 (15.8)	-
IV	55 (9)	1834 (20)	16513 (15.8)	299 (7)
Missing	0 (0)	1222 (13)	-	-
Tumour Type n (%)				
Clear cell	480 (79)	-	* 46818 (72.0)	2424 (75.9)
Papillary	60 (10)	-	8730 (13.4)	435 (13.6)
Chromophobe	46 (8)	-	4127 (6.3)	247 (7.8)
Other RCC	21 (3)	-	5354 (8.2)	87 (2.7)

[a] Shephard E et al. Clinical features of kidney cancer in primary care: a case-control study using primary care records. *Br J Gen Pract* 2013:**63**:e250-5. n=3149 UK cases
[b] Cancer Research UK. https://www.cancerresearchuk.org/health-professional/cancer-statistics-for-the-uk. 2016
[c] http://ncin.org.uk/publications/survival_by_stage. 2017.
[d] Saad et al. Trends in Renal-Cell Carcinoma Incidence and Mortality in the United States in the Last 2 Decades: A SEER-Based Study. Clin Genitourinary Cancer. 2019;**17**:46-57
* amongst 62.2% of cases with known tumour type
[e] Laguna MP, Algaba F, Cadeddu J *et al.* Current patterns of presentation and treatment of renal masses: a clinical research office of the endourological society prospective study. *J Endourol* 2014:**28**:861-70.
Prospective epidemiological, clinical and pathological data on consecutive patients with renal masses treated during a 1-year period in 98 centres worldwide
