# Supplementary material for: Challenges of early renal cancer detection: symptom patterns and incidental diagnosis rate in a multicentre prospective UK cohort of patients presenting with suspected renal cancer
Source: BMJ Open. 2020 May 11;10(5):e035938. doi: 10.1136/bmjopen-2019-035938 (PMC7223292; doi:10.1136/bmjopen-2019-035938)
Supplement: Author's manuscript [file bmjopen-2019-035938.draft_revisions.pdf]

BMJ Open

BMJ Open is committed to open peer review. As part of this commitment we make the peer review history of every article we publish publicly available.

When an article is published we post the peer reviewers' comments and the authors' responses online. We also post the versions of the paper that were used during peer review. These are the versions that the peer review comments apply to.

The versions of the paper that follow are the versions that were submitted during the peer review process. They are not the versions of record or the final published versions. They should not be cited or distributed as the published version of this manuscript.

BMJ Open is an open access journal and the full, final, typeset and author-corrected version of record of the manuscript is available on our site with no access controls, subscription charges or pay-per-view fees (http://bmjopen.bmj.com).

If you have any questions on BMJ Open's open peer review process please email info.bmjopen@bmj.com

BMJ Open

The challenge of early renal cancer detection: symptom patterns and incidental diagnosis rate in a multicentre prospective UK cohort study of patients presenting with suspected renal cancer

Journal:	*BMJ Open*
Manuscript ID	bmjopen-2019-035938
Article Type:	Original research
Date Submitted by the Author:	22-Nov-2019

Complete List of Authors:	Vasudev, Naveen; University of Leeds, Leeds Institute of Medical Research at St James's<br>Wilson, Michelle; University of Leeds, Leeds Institute of Medical Research at St James's<br>Stewart, Grant; University of Cambridge; NHS Lothian<br>Adeyoju, Adebanji; Stockport NHS Foundation Trust<br>Cartledge, Jon; Saint James's University Hospital<br>Kimuli, Michael; Saint James's University Hospital<br>Datta, Shibendra; University Hospital of Wales Healthcare NHS Trust<br>Hanbury, Damian; Lister Hospital<br>Hrouda, David; Charing Cross Hospital<br>Oades, Grenville; Queen Elizabeth University Hospital<br>Patel, Poulam ; University of Nottingham, Division of Cancer and Stem Cell<br>Soomro, Naeem; Newcastle Upon Tyne Hospitals NHS Foundation Trust<br>Sullivan, Mark; Oxford University Hospitals NHS Foundation Trust<br>Webster, Jeff; Northwick Park Hospital<br>Selby, Peter; University of Leeds, Leeds Institute of Medical Research at St James's<br>Banks, Rosamonde; University of Leeds, Leeds Institute of Medical Research at St James's

Keywords:	Kidney tumours < ONCOLOGY, PUBLIC HEALTH, Health policy < HEALTH SERVICES ADMINISTRATION & MANAGEMENT

SCHOLARONE™
Manuscripts

I, the Submitting Author has the right to grant and does grant on behalf of all authors of the Work (as defined in the below author licence), an exclusive licence and/or a non-exclusive licence for contributions from authors who are: i) UK Crown employees; ii) where BMJ has agreed a CC-BY licence shall apply, and/or iii) in accordance with the terms applicable for US Federal Government officers or employees acting as part of their official duties; on a worldwide, perpetual, irrevocable, royalty-free basis to BMJ Publishing Group Ltd ("BMJ") its licensees and where the relevant Journal is co-owned by BMJ to the co-owners of the Journal, to publish the Work in this journal and any other BMJ products and to exploit all rights, as set out in our licence.

The Submitting Author accepts and understands that any supply made under these terms is made by BMJ to the Submitting Author unless you are acting as an employee on behalf of your employer or a postgraduate student of an affiliated institution which is paying any applicable article publishing charge ("APC") for Open Access articles. Where the Submitting Author wishes to make the Work available on an Open Access basis (and intends to pay the relevant APC), the terms of reuse of such Open Access shall be governed by a Creative Commons licence – details of these licences and which Creative Commons licence will apply to this Work are set out in our licence referred to above.

Other than as permitted in any relevant BMJ Author's Self Archiving Policies, I confirm this Work has not been accepted for publication elsewhere, is not being considered for publication elsewhere and does not duplicate material already published. I confirm all authors consent to publication of this Work and authorise the granting of this licence.

For peer review only

The challenge of early renal cancer detection: symptom patterns and incidental diagnosis rate in a multicentre prospective UK cohort study of patients presenting with suspected renal cancer

Naveen S. Vasudev[1], Michelle Wilson[1], Grant D. Stewart[2*], Adebanji Adeyoju[3], Jon Cartledge[4], Michael Kimuli[4], Shibendra Datta[5], Damian Hanbury[6], David Hrouda[7], Grenville Oades[8], Poulam Patel[9], Naeem Soomro[10], Mark Sullivan[11], Jeff Webster[12], Peter J. Selby[1], Rosamonde E. Banks[1]

[1]Leeds Institute of Medical Research at St James's, St. James's University Hospital, Beckett Street, Leeds LS9 7TF, UK.
[2]NHS Lothian, Edinburgh, UK.
[3]Stockport NHS Foundation Trust, Stockport, UK.
[4]Department of Urology, St James's University Hospital, Beckett Street, Leeds LS9 7TF, UK.
[5]University Hospital of Wales, Cardiff Heath Park, Cardiff, Wales CF14 4XW
[6]Lister Hospital, Stevenage SG1 4AB UK
[7]Charing Cross Hospital, Imperial College Healthcare NHS Trust, London W6 8RF
[8]Queen Elizabeth University Hospital, Glasgow G51 4TF UK
[9]Division of Cancer & Stem Cells, School of Medicine, University of Nottingham, Nottingham UK
[10]Newcastle Upon Tyne Hospitals NHS Foundation Trust, Newcastle upon Tyne, UK
[11]Oxford University Hospitals NHS Foundation Trust, Oxford UK
[12]Northwick Park Hospital Watford Road Harrow, HA1 3UJ UK
*Current affiliation: Department of Surgery, University of Cambridge, Cambridge Biomedical Campus, Addenbrooke's Hospital, Cambridge, CB2 0QQ, UK

Corresponding author: Dr N Vasudev, St. James's University Hospital, Beckett Street, Leeds LS9 7TF, UK. Tel: +44 113 2067680; Fax: +44 113 2429886; e-mail: n.vasudev@leeds.ac.uk

Key words: Diagnosis; Haematuria; Incidental; Presentation; Renal cell carcinoma; Symptoms

For peer review only - http://bmjopen.bmj.com/site/about/guidelines.xhtml

Abstract

Objectives: To describe the frequency and nature of symptoms in patients presenting with suspected renal cell carcinoma (RCC) and examine their reliability in achieving early diagnosis

Design: Multicentre prospective observational cohort study

Setting and Participants: Eleven UK centres recruiting patients presenting with suspected newly diagnosed RCC. Symptoms reported by patients were recorded and reviewed. Comprehensive clinico-pathological and outcome data were also collected.

Outcomes: Type and frequency of reported symptoms. Incidental diagnosis rate. Metastasis-free and cancer-specific survival.

Results: From 706 patients recruited between 2011-2014, 608 patients with a confirmed RCC formed the primary study population. The majority (60%) of patients were diagnosed incidentally. 87% patients with stage Ia and 36% with stage III or IV disease presented incidentally. Visible haematuria was reported in 23% of patients and was commonly associated with advanced disease (49% had stage III or IV disease). Symptomatic presentation was associated with poorer outcomes. Symptom patterns amongst the 54 patients subsequently found to have a benign renal mass were similar to those with a confirmed RCC.

Conclusions: Raising public awareness of RCC-related symptoms as a strategy to improve early detection rates is limited by the fact that related symptoms are relatively uncommon and often associated with advanced disease. Greater attention must be paid to the feasibility of screening strategies and the identification of circulating diagnostic markers.

For peer review only

Strengths and limitations of this study

- The multicentre, prospective nature of this study, amongst a contemporary cohort of UK patients, is unique and represents an important strength over previous studies
- Comprehensive linked clinico-pathological and outcome data was available for all patients
- Symptoms amongst patients subsequently found to have a benign renal mass are reported in parallel
- Patient reported symptoms were recorded following referral to secondary care and may therefore be subject to recall bias

For peer review only

For peer review only - http://bmjopen.bmj.com/site/about/guidelines.xhtml

Introduction

The incidence of kidney cancer in Europe is amongst the highest worldwide. In the UK, incidence rates have risen by 47% increase over the past decade, with 12,000 new cases in 2015 [1]. By 2035, it is predicted that this number will rise to over 20,000 new cases per annum and kidney cancer will come to represent the 4th commonest cancer amongst males and 9th commonest amongst females in the UK [2].

Diagnosing patients with kidney cancer can be challenging [3]. Renal cell carcinomas (RCCs), which make up the majority (85%) of kidney cancers, are characteristically insidious in onset. The once classical triad of haematuria, pain and abdominal mass is now recognised to be rare and symptoms, if present at all, can be vague, non-specific and delayed in onset. Whilst early diagnosis is recognised to be key in achieving optimal outcomes, many patients still present with advanced disease. In 2017 in England, for example, figures show that amongst patients with a recorded stage at diagnosis, 19% had stage III and 23% had stage IV disease, at the time of presentation [4].

Campaigns to raise awareness of kidney cancer amongst the public and doctors have been employed in an effort to improve early diagnosis rates [5]. Understanding how patients present may help to inform such strategies. Unlike previous studies, we prospectively collected information on symptoms reported by patients at the time of their diagnosis of suspected RCC, following recruitment to a large, contemporary, multi-institutional UK RCC biobank [6]. We describe symptoms reported by patients and define the current rate of incidental diagnosis with the goal of better understanding the challenges in early RCC diagnosis.

For peer review only - http://bmjopen.bmj.com/site/about/guidelines.xhtml

Methods

The design was a multicentre prospective observational cohort study. Details regarding the inclusion and exclusion criteria are as previously reported [6]. Comprehensive clinical and pathological information was collected.

At the time of recruitment to the study, patients were asked about the presence and nature of symptoms leading to their diagnosis of suspected RCC, which was recorded using paper case-report forms (CRF). Specific questions relating to commonly related 'RCC-type' local symptoms (pain, haematuria, abdominal mass and/or other) and/or systemic symptoms (weight loss (any), loss of appetite, sweats, fevers, fatigue and/or other) were recorded. In addition, the investigator completing the CRF was asked to state whether the diagnosis was incidental in nature and included a subsequent free-text box requesting a description of how the patient was diagnosed. All cases were independently reviewed by two reviewers (NV and RB) to confirm or refute whether the diagnosis would be regarded as incidental or not (i.e. were any symptoms reported and, if so, would they be regarded as being related to the finding of RCC), with additional reference to individual electronic case notes where available. Reported presence of RCC-type symptoms, many of which are non-specific, was not always related to the finding of RCC. Cases with insufficient data or where the incidental nature of the diagnosis remained uncertain were not classified. Patients being investigated for asymptomatic hypertension were not classified as incidental [7].

Metastasis-free survival (MFS) was calculated for patients with localised disease, defined as the period from date of nephrectomy to date of distant recurrence.

For peer review only - http://bmjopen.bmj.com/site/about/guidelines.xhtml

Patients without recurrence were censored at the date they were last known to be recurrence-free (for patients who died without recurrence this was date of death). Cancer-specific survival (CSS) was defined as the period from date of nephrectomy to the date of cancer-related death. Patients with a non-cancer related death were censored at their date of death and patients still alive were censored at the last date they were known to be alive. Kaplan-Meier plots were produced to visualise survival and the log-rank test was used to detect statistically significant difference between survival curves.

For peer review only

For peer review only - http://bmjopen.bmj.com/site/about/guidelines.xhtml

Results

Between July 2011 and June 2014, 706 patients were recruited to the study from 11 UK centres. RCC was confirmed in 608 (86%) patients, amongst whom median follow-up was 4.8 yrs (IQR: 3.7, 5.2), and benign renal mass in 54 (7.6%) patients. The remaining 44 (6.4%) patients either did not undergo biopsy or nephrectomy or had no tumour in their biopsy cores (n=33), had another (not RCC) malignancy (n=5), or an alternative benign pathology (n=6) [6]. Amongst all patients with a confirmed RCC, 422 (69%) patients reported having RCC-type symptoms at diagnosis, of whom 221 (52%) reported symptoms that were considered related to the presence of RCC. Amongst these 221 patients, 97 (44%) had local symptoms only, 19 (8.6%) had systemic symptoms only and 105 (47.5%) reported having both local and systemic symptoms. Patient and tumour characteristics by symptom type are shown in **Table 1**.

Local RCC-related symptoms

Amongst the 202 (33%) patients reporting local RCC-related symptoms, 137 (68%) reported visible haematuria and 126 (62%) reported pain, with only 14 (7%) patients reporting an abdominal mass. Patients presenting with haematuria had a median pathological tumour size of 75mm (range 16-155) and almost half had stage III (37.2%) or IV (12.4%) disease. Only four patients (0.6%) presented with the classical triad of an abdominal mass, haematuria and local pain. The median tumour size amongst these four patients was 105 mm (range 80-154 mm) on preoperative cross-sectional imaging.

For peer review only - http://bmjopen.bmj.com/site/about/guidelines.xhtml

Systemic RCC-related symptoms

Amongst those reporting systemic symptoms related to their RCC, fatigue (62%), weight loss (52%), sweats (38%) and loss of appetite (38%) were all commonly reported. Fever was relatively uncommon (10%). Patients with systemic symptoms were more likely to have grade 4 cancers and stage IV disease than those with local RCC-related symptoms only and those with symptoms unrelated to RCC ($p<0.01$) (**Table 1**).

Incidental diagnosis

Amongst the 582 patients in whom the nature of the diagnosis could be confidently classified, 351 (60%) cases of RCC were deemed to have been diagnosed incidentally. Patient and tumour characteristics by nature of diagnosis (incidental vs non-incidental) are shown in **Table 2**. No association with patient sex was found and distribution of histological subtype was similar between groups. Non-incidentally detected tumours were larger and of higher grade and stage than incidentally detected tumours ($p<0.01$). Amongst patients diagnosed with a localised pT1a tumour, the incidental diagnosis rate was 87%. Conversely, 22% of patients with stage IV disease were considered to have been diagnosed incidentally. The nature of the incidental diagnosis (e.g. during investigation for a known pre-existing condition versus investigation of unrelated symptoms) is shown in **Table 3**.

Tumour size

Pathological tumour size was available for 556 (91%) of patients. We looked at symptoms in patients presenting with tumours ≥10 cm. Amongst the 66 patients with a tumour ≥10cm, 31 (47%) reported haematuria at the time of presentation, 33 (50%)

For peer review only - http://bmjopen.bmj.com/site/about/guidelines.xhtml

reported pain, and abdominal mass was reported in four (6%) patients. Almost a quarter (16/66; 24%) of these patients were considered to have been diagnosed incidentally, with 10 (15%) reporting no symptoms, despite the presence of a large primary tumour. No effect of BMI was observed in relation to presence or absence of symptoms.

Outcomes

We looked at survival outcomes by both symptom type (no RCC-type symptoms or unrelated RCC-type symptoms vs. related RCC-type symptoms) and incidental versus non-incidental diagnosis. Patients diagnosed with no RCC-type symptoms and those reporting unrelated RCC-type symptoms had a significantly improved MFS and CSS compared to patients with related RCC-type symptoms. Furthermore, patients with systemic RCC-related symptoms had poorer outcomes than those with local RCC symptoms only (**Figure 1 A and B**). Overall, patients with an incidental diagnosis of RCC had improved MFS and CSS in comparison to those diagnosed non-incidentally, although these effects were mostly lost when controlled for stage of disease (**Figure 2**).

Patients presenting with benign renal masses

In total, 54 (7.6%) patients in our cohort were found to have a benign renal mass, composed of oncocytoma (n=29), angiomyolipoma (n=8) and other lesions (n=17) (**Table 4**). The incidental diagnosis rate was 56% amongst the 52 evaluable patients. Haematuria and pain were reported in 57% and 52% of patients diagnosed non-incidentally. The majority (65%) reported symptoms, of whom 57% had local symptoms only, 17% had systemic symptoms only and 26% reported both local and systemic symptoms.

For peer review only - http://bmjopen.bmj.com/site/about/guidelines.xhtml
For peer review only

Discussion

Early detection is widely held to be a key strategy towards improving outcomes in patients with RCC [8]. As in most solid cancers, disease stage and survival are closely linked, with 3-year CSS rates in our cohort, for example, of 99% and 47% for stage I and stage IV cancers, respectively (data not shown). Symptoms of kidney cancer such as visible haematuria and flank pain are well documented and NHS initiatives such as 'be clear on cancer: blood in your pee' campaign have been aimed at prompting the public to seek early medical attention [5]. Nevertheless, many patients still present with overt or micro-metastatic disease. Understanding the type and frequency of symptoms patients with newly diagnosed RCC report is critical in beginning to address this issue and understand whether simply raising awareness amongst doctors and the public is sufficient or other strategies are needed.

Our study highlights the significant challenges in diagnosing patients with kidney cancer. Almost a third of patients in our cohort were symptomless at the time of diagnosis, amongst whom nearly a quarter (24%) had stage III or IV disease. Visible haematuria, a hallmark symptom of this disease, was recorded in just 23% of patients overall. Even amongst patients with large (≥10cm) tumours, less than half (47%) reported haematuria as a symptom. Prior reports using UK general practice database records have suggested rates of haematuria as low as 18% in patients presenting with kidney cancer, compounded by the low positive predictive value (PPV) (1%) of this symptom for RCC amongst those ≥60 yr old [9]. Furthermore, symptom patterns do not appear to reliably distinguish patients with benign renal masses from those with RCC.

For peer review only - http://bmjopen.bmj.com/site/about/guidelines.xhtml

Many studies have attempted to document the incidental diagnosis rate for renal cancer. These previous studies have all been retrospective in nature, typically derived from patients at a single centre, with widely varying rates of incidental diagnosis, from 15% to 61%, in a less contemporaneous setting (broadly spanning 1970-2000) [10-14]. A more recent, global, study, involving 4288 patients presenting with RCC between 2010-2012, reported an incidental diagnosis rate of 67%, however no detail regarding how this was derived, or the nature and characteristics of those diagnosed incidentally were presented in this study [15]. We carefully reviewed the presenting symptoms and history for each patient in our study, performed independently by two experts, to determine as accurately as possible whether the diagnosis would be deemed incidental or not. Pain, for example, was a commonly reported symptom not necessarily attributable to the diagnosis of RCC, for example when located in an anatomically distinct site. We believe our figure of 60%, amongst a contemporary set of patients (2011-2014), provides a true reflection of the current incidental diagnosis rate of RCC in the UK, and supports the general rise in the incidental detection of kidney cancer that has been reported over time.

Our data shows that the majority (60%) of patients with RCC in the UK are being diagnosed incidentally, in most cases (74%) during investigation of symptoms unrelated to RCC. By contrast, a Norwegian study of 413 patients diagnosed with RCC between 1997-2010 reported a 53% incidental diagnosis rate, detected in 63% of these patients during follow-up for a pre-existing condition [16]. The reason for this difference is not certain but may reflect the different time periods under study, given the more liberal use of cross-sectional imaging over time [17]. Consistent with other studies, patients with an incidentally detected RCC tended to have smaller, lower

For peer review only - http://bmjopen.bmj.com/site/about/guidelines.xhtml

stage and grade tumours than those presenting with related symptoms, but, nevertheless, almost one in five of patients identified incidentally had stage III/IV disease at diagnosis. Whether patients who are diagnosed incidentally have better outcomes and potentially, therefore, different tumour biology, than those presenting with symptoms has been a matter of debate in the literature [10,18-20]. We did not find any difference in MFS or CSS between these two groups when matched for stage of disease, suggesting that incidental detection of advanced stage disease is not advantageous in terms of outcome.

Diagnosing kidney cancer early is therefore a significant public health challenge. Data from the 2010 National Cancer Patient Experience Survey in England report that almost 30% of 564 patients with renal cancer saw their general practitioner three or more times before hospital referral [21]. Furthermore, results from the charity Kidney Cancer UK (KCUK) 2018 patient survey showed that 22% of the 153 responders who presented to their GP or an A+E department waited more than 3 months for a diagnosis [22]. The results of the KCUK survey (n=175 in total) extend further, with 51% of patients reporting their cancer being detected incidentally during imaging for an unrelated reason, and less than one third (31%) having symptoms due to RCC, reflecting the findings from our own, much larger, study.

How then do we improve rates of early diagnosis in kidney cancer? Raising awareness amongst the public to present early to their doctor, even with vague symptoms, may seem logical, as well as increasing awareness with primary care teams. But many patients remain asymptomatic until they have advanced stage disease, and the PPVs for symptoms other than haematuria, such as pain and

For peer review only - http://bmjopen.bmj.com/site/about/guidelines.xhtml

fatigue, are even lower than 1% [9], placing an impossible demand on general practitioners, who are required to act as gatekeepers to secondary care. Five-year survival rates for kidney cancer in the UK lag behind the European average which may be related to differences in stage at diagnosis [23]. Greater availability of point-of-care ultrasound may make a significant impact but its use varies widely across Europe and has not been widely adopted in the UK, with potential barriers in terms of time and training [24].

Interest in exploring the potential for kidney cancer screening is growing [8,25], particularly given the significant predicted rise in incidence [2]. The potential cost-effectiveness of performing a single, renal focused, USS amongst asymptomatic 60-year-old men has recently been reported [26]. However numerous uncertainties still exist, in terms of who to screen, with what modality, as well as unknowns in terms of associated harms versus benefit [27]. This is an area that clearly warrants further research. The identification of robust diagnostic biomarkers either in the serum or urine of patients that could be used to easily rule in or out the presence of RCC is another priority area for study [28], with recent promising reports in the literature [29], although still requiring significant further validation and improved performance.

In summary, this study draws attention to the fact that reliance on symptoms for the early detection of kidney cancer is not robust. Our data suggest that improving public and professional awareness will have only a limited impact, and innovative biomarkers for this purpose remain to be identified. We suggest it is time to re-examine the case for screening looking at opportunities to link RCC screening into

For peer review only - http://bmjopen.bmj.com/site/about/guidelines.xhtml
For peer review only

other programmes such as low dose CT scans for lung cancer health checks or ultrasound-based screening for abdominal aortic aneurysms.

Contributors: Funding: PJS and REB; Study design and concept: PJS, REB, NSV; Patient recruitment and data collection: AA, JC, MK, SD, DHa, DHr, GO, PP, NS, GDS, MS, JW, NSV, REB; Data analysis: MW, GDS, NSV, REB; Manuscript preparation: NSV, MW, GDS, PJS, REB. All authors participated in the interpretation of results, critically revised the paper and approved the final version to be published.

Ethics approval and consent to participate: The study was approved by the Leeds East Research Ethics Committee (ethical approval 10/H1306/6). Patients were recruited to the study with their written informed consent

Funding: This project was funded by the National Institute for Health Research Programme Grants for Applied Research programme (project number NIHR PGfAR RP-PG-0707-10101) and has been published in full in Programme Grants for Applied Research Volume: 6, Issue: 3. Further information is available at: https://www.journalslibrary.nihr.ac.uk/pgfar/pgfar06030/#/abstract. Additional support was provided by the NIHR Leeds Clinical Research Facility. The views and opinions expressed by the authors in this publication are those of the authors and do not necessarily reflect those of the NHS, the NIHR or the Department of Health.

Disclaimer: The funder had no role in study design, data collection, data analysis or manuscript preparation

Patient and public involvement: Patients were extensively involved in the design, delivery and evaluation of the NIHR Programme supporting this work. Patients were not directly involved in the design or evaluation of the current report.

Conflicts of interest: N.S.V. discloses paid consultancy work and/or speaker honoraria for EUSA pharma, Bristol Myers Squibb, Pfizer, Merck and Novartis. G.D.S. discloses paid consultancy work for Pfizer, EUSA Pharma, Merck, and CMR Surgical and an advisory board role for the latter. The other authors declare no conflicts of interest.

Acknowledgements: We are grateful to the patients, the staff at participating centres who assisted in patient recruitment, staff within the Leeds Biobanking and Sample Processing Lab and staff at the Leeds Clinical Trials Research Unit.

Patient consent for publication: Not required

Data availability statement: All data relevant to the study are included in the article

For peer review only

References

1. Cancer Research UK. https://www.cancerresearchuk.org/health-professional/cancer-statistics-for-the-uk. Accessed August 2019.
2. Smittenaar CR, Petersen KA, Stewart K, Moitt N. Cancer incidence and mortality projections in the UK until 2035. *Br J Cancer* 2016:**115**:1147-55
3. Zhou Y, van Melle M, Singh H, Hamilton W, Lyratzopoulos G, Walter FM. Quality of the diagnostic process in patients presenting with symptoms suggestive of bladder or kidney cancer: a systematic review. *BMJ Open* 2019:**9**:e029143
4. http://ncin.org.uk/publications/survival_by_stage. Accessed August 2019
5. https://www.nhs.uk/be-clear-on-cancer/symptoms/blood-in-pee. Accessed August 2019
6. Vasudev NS, Hutchinson M, Trainor S *et al.* UK multicentre prospective evaluation of the Leibovich score in localised renal cell carcinoma: performance has altered over time. *Urology* 2019. In Press
7. Chow WH, Gridley G, Fraumeni JF, Jr., Jarvholm B. Obesity, hypertension, and the risk of kidney cancer in men. *N Engl J Med* 2000:**343**:1305-11
8. Motzer RJ. Perspective: What next for treatment? *Nature* 2016:**537**:S111
9. Shephard E, Neal R, Rose P, Walter F, Hamilton WT. Clinical features of kidney cancer in primary care: a case-control study using primary care records. *Br J Gen Pract* 2013:**63**:e250-5
10. Tsui KH, Shvarts O, Smith RB, Figlin R, de Kernion JB, Belldegrun A. Renal cell carcinoma: prognostic significance of incidentally detected tumors. *J Urol* 2000:**163**:426-30
11. Lee CT, Katz J, Fearn PA, Russo P. Mode of presentation of renal cell carcinoma provides prognostic information. *Urol Oncol* 2002:**7**:135-40
12. Ficarra V, Prayer-Galetti T, Novella G *et al.* Incidental detection beyond pathological factors as prognostic predictor of renal cell carcinoma. *Eur Urol* 2003:**43**:663-9
13. Luciani LG, Cestari R, Tallarigo C. Incidental renal cell carcinoma-age and stage characterization and clinical implications: study of 1092 patients (1982-1997). *Urology* 2000:**56**:58-62
14. Jayson M, Sanders H. Increased incidence of serendipitously discovered renal cell carcinoma. *Urology* 1998:**51**:203-5
15. Laguna MP, Algaba F, Cadeddu J *et al.* Current patterns of presentation and treatment of renal masses: a clinical research office of the endourological society prospective study. *J Endourol* 2014:**28**:861-70
16. Sand KE, Hjelle KM, Rogde AJ, Gudbrandsdottir G, Bostad L, Beisland C. Incidentally detected renal cell carcinomas are highly associated with comorbidity and mortality unrelated to renal cell carcinoma. *Scand J Urol* 2013:**47**:462-71
17. https://www.gov.uk/government/publications/review-of-radiation-dose-issues-from-the-use-of-ct-in-the-uk. Accessed August 2019
18. Patard JJ, Rodriguez A, Rioux-Leclercq N, Guille F, Lobel B. Prognostic significance of the mode of detection in renal tumours. *BJU Int* 2002:**90**:358-63
19. Palsdottir HB, Hardarson S, Petursdottir V *et al.* Incidental detection of renal cell carcinoma is an independent prognostic marker: results of a long-term, whole population study. *J Urol* 2012:**187**:48-53

For peer review only - http://bmjopen.bmj.com/site/about/guidelines.xhtml

20. Gudbjartsson T, Thoroddsen A, Petursdottir V, Hardarson S, Magnusson J, Einarsson GV. Effect of incidental detection for survival of patients with renal cell carcinoma: results of population-based study of 701 patients. *Urology* 2005:**66**:1186-91
21. Lyratzopoulos G, Neal RD, Barbiere JM, Rubin GP, Abel GA. Variation in number of general practitioner consultations before hospital referral for cancer: findings from the 2010 National Cancer Patient Experience Survey in England. *Lancet Oncol* 2012:**13**:353-65
22. https://www.kcuk.org.uk/patient-survey-2018/. Accessed August 2019
23. De Angelis R, Sant M, Coleman MP *et al.* Cancer survival in Europe 1999-2007 by country and age: results of EUROCARE--5-a population-based study. *Lancet Oncol* 2014:**15**:23-34
24. Mengel-Jorgensen T, Jensen MB. Variation in the use of point-of-care ultrasound in general practice in various European countries. Results of a survey among experts. *Eur J Gen Pract* 2016:**22**:274-7
25. Shea MW. A proposal for a targeted screening program for renal cancer. *Front Oncol* 2013:**3**:207
26. Rossi SH, Klatte T, Usher-Smith JA *et al.* A Decision Analysis Evaluating Screening for Kidney Cancer Using Focused Renal Ultrasound. *Eur Urol Focus* 2019
27. Rossi SH, Klatte T, Usher-Smith J, Stewart GD. Epidemiology and screening for renal cancer. *World J Urol* 2018:**36**:1341-53
28. Capitanio U, Bensalah K, Bex A *et al.* Epidemiology of Renal Cell Carcinoma. *Eur Urol* 2019:**75**:74-84
29. Morrissey JJ, Mellnick VM, Luo J *et al.* Evaluation of Urine Aquaporin-1 and Perilipin-2 Concentrations as Biomarkers to Screen for Renal Cell Carcinoma: A Prospective Cohort Study. *JAMA Oncol* 2015:**1**:204-12

For peer review only

For peer review only - http://bmjopen.bmj.com/site/about/guidelines.xhtml

Figure Legends

Figure 1. Kaplan Meier survival curves by symptom type. Survival outcomes (A. MFS; B. CSS) in patients with no RCC-type symptoms, unrelated RCC-type symptoms, local RCC-related symptoms and those with systemic (+/- local) RCC-related symptoms

Figure 2. Kaplan Meier survival curves by incidental vs. non-incidental diagnosis for all patients, stage I/II or stage III RCC. A-C: MFS; D-F: CSS

For peer review only

For peer review only - http://bmjopen.bmj.com/site/about/guidelines.xhtml

Table 1. Patient and tumour characteristics by symptom type
For continuous variables, figures in table represent median (range) with corresponding p-value from the Kruskal-Wallis test and for categorical variables, figures in table represent n (%) with corresponding p-value from chi-squared test.

		RCC-type symptoms reported (n=422)**			
**Characteristic**	**No RCC-type symptoms (n=186)**	**Not RCC related (n=183)**	**RCC-related local symptoms only (n=97)**	**RCC-related systemic symptoms (+/- local) (n=124)**	**p-value**
**Age (years)**	65 (31-86)	63 (29-90)	63 (38-84)	62 (33-92)	0.31
**Gender**					
**Female**	67 (32.7)	62 (30.2)	21 (10.2)	55 (26.8)	
**Male**	119 (30.9)	121 (31.4)	76 (19.7)	69 (17.9)	0.01
**BMI**	28.5 (15.6-74.4)	27 (18.1-56.5)	28.8 (17.3-67.2)	27.5 (16-54.5)	0.01
**Tumour size (mm)**	44 (14-180)	43 (11-170)	74 (13-155)	75 (20-240)	<0.01
**pT**					
**1a**	83 (42.6)	88 (45.1)	16 (8.2)	8 (4.1)	
**1b**	46 (34.3)	42 (31.3)	19 (14.2)	27 (20.1)	
**2**	15 (19.7)	18 (23.7)	19 (25)	24 (31.6)	
**3**	38 (22.6)	33 (19.6)	42 (25)	55 (32.7)	
**4**	0 (0)	0 (0)	1 (25)	3 (75)	
**X**	1 (50)	1 (50)	0 (0)	0 (0)	
**Missing**	1 (100)	0 (0)	0 (0)	0 (0)	
**NA**	2 (20)	1 (10)	0 (0)	7 (70)	<0.01
**Grade**					
**1**	4 (40)	0 (0)	4 (40)	2 (20)	
**2**	55 (34.8)	50 (31.6)	25 (15.8)	28 (17.7)	
**3**	88 (32.2)	94 (34.4)	47 (17.2)	44 (16.1)	
**4**	13 (14.9)	13 (14.9)	19 (21.8)	42 (48.3)	
**Missing**	9 (39.1)	9 (39.1)	1 (4.3)	4 (17.4)	
**NA**	17 (43.6)	17 (43.6)	1 (2.6)	4 (10.3)	<0.01
**Stage**					
**I**	130 (39.8)	129 (39.4)	34 (10.4)	34 (10.4)	
**II**	12 (17.4)	17 (24.6)	18 (26.1)	22 (31.9)	
**III**	34 (24.5)	29 (20.9)	37 (26.6)	39 (28.1)	
**IV**	10 (18.9)	6 (11.3)	8 (15.1)	29 (54.7)	
**Missing**	0 (0)	2 (100)	0 (0)	0 (0)	<0.01
**Tumour subtype**					
**Clear Cell**	147 (31.7)	137 (29.6)	83 (17.9)	96 (20.7)	
**Papillary**	16 (27.1)	23 (39)	7 (11.9)	13 (22)	
**Chromophobe**	15 (32.6)	15 (30.4)	7 (15.2)	10 (21.7)	
**Unclassified**	7 (38.9)	6 (33.3)	0 (0)	5 (27.8)	
**Other**	1 (33)	2 (67)	0 (0)	0 (0)	0.81

*NA=not applicable - patients underwent biopsy only or tumour ablation
**18 patients reported symptoms but their relationship to RCC could not be determined

For peer review only

Table 2. Patient and tumour characteristics by diagnosis type
For continuous variables, figures in table represent median (range) with corresponding p-value from the Wilcoxon rank-sum test and for categorical variables, figures in table represent n (%) with corresponding p-value from chi-squared test.

Characteristic	Non-incidental (n=231)	Incidental (n=351)	p-value
**Age (years)**	62 (33-92)	65 (29-90)	0.04
**Gender**			
**Female**	77 (38.3)	124 (61.7)	
**Male**	154 (40.4)	227 (59.6)	0.69
**BMI**	28.3 (15.6-67.2)	27.8 (17.2-57.7)	0.38
**Tumour size (path) (mm)**	75 (13-240)	42 (11-170)	<0.01
**Tumour size (CT) (mm)**	80 (16-250)	44 (10-170)	<0.01
**pT**			
**1a**	25 (12.8)	170 (87.2)	
**1b**	48 (37.2)	81 (62.8)	
**2**	46 (60.5)	30 (39.5)	
**3**	101 (61.2)	64 (38.8)	
**4**	4 (100)	0 (0)	
**X**	0 (0)	2 (100)	
**Missing**	0 (0)	1 (100)	
**NA***	7 (70)	3 (30)	<0.01
**Grade**			
**1**	6 (66.7)	3 (33.3)	
**2**	56 (35.9)	100 (64.1)	
**3**	93 (34.6)	176 (65.4)	
**4**	65 (75.6)	21 (24.4)	
**Missing**	6 (26.1)	17 (73.9)	
**NA***	5 (12.8)	34 (87.2)	<0.01
**Stage**			
**I**	70 (21.6)	254 (78.4)	
**II**	42 (60.9)	27 (39.1)	
**III**	80 (58.4)	57 (41.6)	
**IV**	39 (78)	11 (22)	
**Missing**	0 (0)	2 (100)	<0.01
**Tumour subtype**			
**Clear Cell**	186 (40.9)	269 (59.1)	
**Papillary**	21 (35.6)	38 (64.4)	
**Chromophobe**	19 (41.3)	28 (58.7)	
**Unclassified**	5 (27.8)	13 (72.2)	
**Other**	0 (0)	3 (100)	0.62

*NA=not applicable, patients underwent biopsy only or tumour ablation

For peer review only - http://bmjopen.bmj.com/site/about/guidelines.xhtml

Table 3. Nature of incidental diagnosis

Type of incidental diagnosis	n (%)
**Investigation for pre-existing condition**	65 (18)
Another malignancy	34 (53)
Diabetes Mellitus	7 (11)
Hepatobiliary[a]	5 (8)
AAA screening / Post-aortic repair	3 (5)
Other[b]	16 (23)
**Investigation for signs or symptoms unrelated to RCC**	258 (74)
Gastrointestinal[c]	86 (33)
Urinary tract[d]	49 (19)
Hepatobiliary[e]	27 (10)
Respiratory[f]	20 (8)
Musculoskeletal[g]	16 (6)
Cardiovascular[h]	11 (4)
Trauma	7 (3)
Gynaecological	6 (3)
Anaemia	4 (2)
Miscellaneous[i]	32 (12)
**Routine health check**[k]	16 (5)
**Not known**[l]	12 (3)

AAA, abdominal aortic aneurysm; [a]cirrhosis, primary biliary cirrhosis, sclerosing cholangitis; [b]includes Addison's disease, chronic renal failure, crohn's disease, coeliac disease, ovarian cyst, renal stones, IgA nephropathy, Wegener's granulomatosis, polymyalgia rheumatica, ovarian cyst; [c]altered bowel habit, GI bleed, bloating/distension, abdominal pain, reflux; [d]urinary retention, prostatic symptoms, high PSA, urosepsis, renal colic, impaired renal function; [e]biliary colic, deranged liver function tests, jaundice, pancreatitis, cholecystitis; [f]shortness of breath, cough, haemoptysis, pneumonia; [g]back pain, leg pain, joint pain; [h]chest pain, myocardial infarction, claudication, endocarditis; [i]includes dizziness, syncope, elevated blood test values, ankle swelling; [k]Initial investigations were urine dip (6), USS (5), CT scan (2), blood tests (2), CXR (1); [l]insufficient information to classify

For peer review only

For peer review only - http://bmjopen.bmj.com/site/about/guidelines.xhtml

Table 4. Characteristics and symptoms associated with benign renal masses

Characteristic	All (n=54)	Oncocytoma (n=29)	AML (n=8)	Other* (n=17)
**Age (years)**	65 (32-86)	66 (42-86)	63 (59-68)	61 (32-78)
**Gender**				
**Female**	29 (53.7)	12 (41.4)	5 (62.5)	12 (70.6)
**Male**	25 (46.3)	17 (58.6)	3 (37.5)	5 (29.4)
**BMI**	27.6 (18.7-45.8)	27.8 (19.4-39.6)	28 (22-38.8)	26.4 (18.7-45.8)
**CT size (cm)**				
**≤4**	22 (44.9)	14 (50)	3 (50)	5 (33.3)
**4< - ≤7**	18 (36.7)	11 (39.3)	2 (33.3)	5 (33.3)
**7< - ≤10**	6 (12.2)	1 (3.6)	1 (16.7)	4 (26.7)
**>10**	3 (6.1)	2 (7.1)	0 (0)	1 (6.7)
**NA**	5 (-)	1 (-)	2 (-)	2 (-)
**RCC-type symptoms**				
**No**	19 (35.2)	10 (34.5)	4 (50)	5 (29.4)
**Yes**	35 (64.8)	19 (65.5)	4 (50)	12 (70.6)
**Local symptoms**				
**No**	6 (17.1)	3 (15.8)	1 (25)	2 (16.7)
**Yes**	29 (82.9)	16 (84.2)	3 (75)	10 (83.3)
**Systemic symptoms**				
**No**	20 (57.1)	12 (63.2)	1 (25)	7 (58.3)
**Yes**	15 (42.9)	7 (36.8)	3 (75)	5 (41.7)
**Incidental diagnosis**				
**No**	23 (42.5)	13 (44.8)	2 (25)	8 (47)
**Yes**	29 (54)	15 (51.7)	6 (75)	8 (47)
**Not known**	2 (3.5)	1 (3.5)	0 (0)	1 (6)

AML – angiomyolipoma *consists of cystic nephroma (4), benign cyst (3), metanephric adenoma (2), mixed epithelial stromal tumour (2), haemangioblastoma (1), leiomyomata (1), multilocular cyst (1), myxoid mesenchymal tumour (1), Rosai Dorfman disease (1), solitary fibrous tumour (1)

For peer review only

For peer review only - http://bmjopen.bmj.com/site/about/guidelines.xhtml

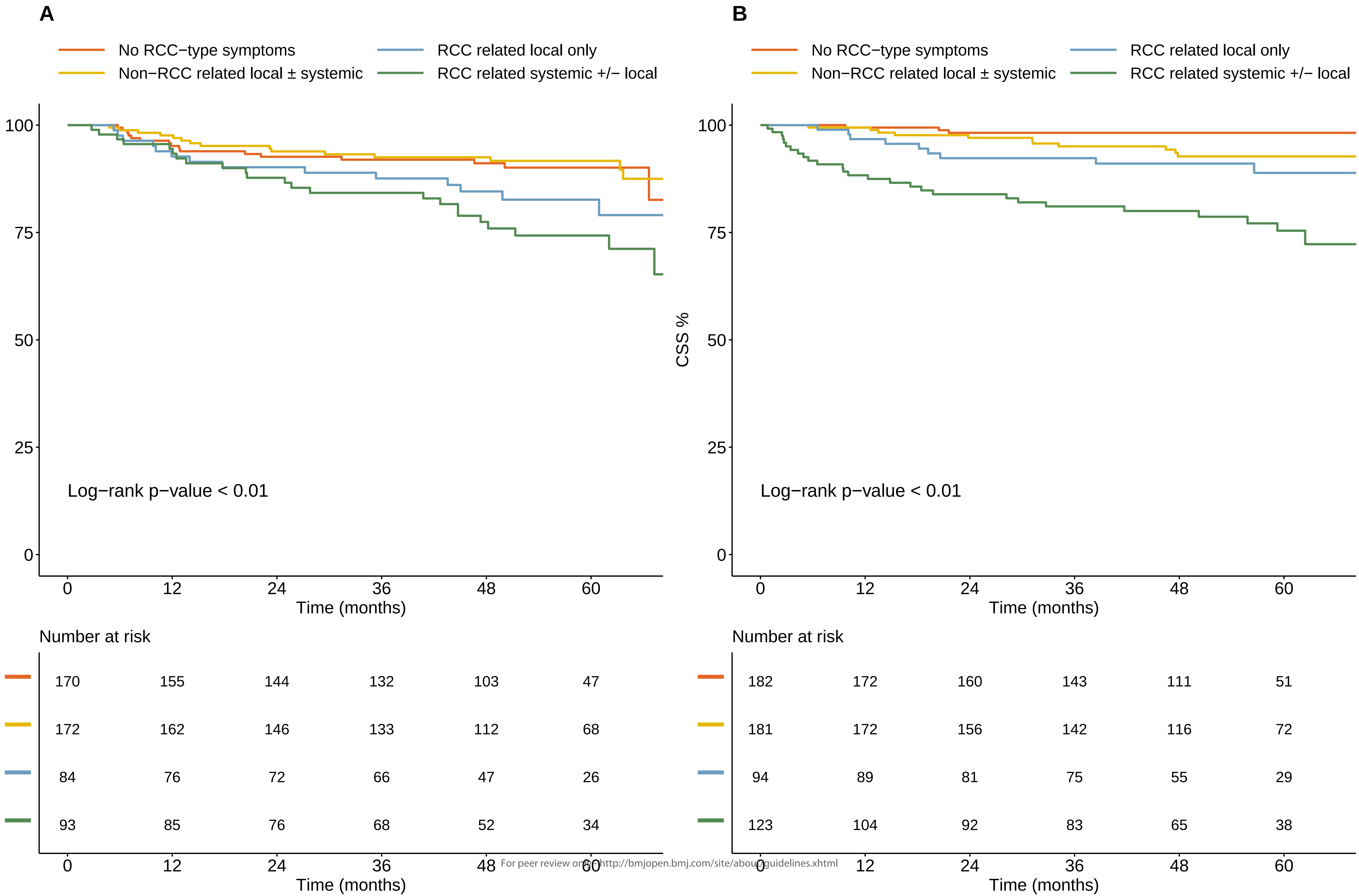
A
No RCC−type symptoms
Non−RCC related local ± systemic
RCC related local only
RCC related systemic +/− local
MFS %
100
75
50
25
0
Log−rank p−value < 0.01
0 12 24 36 48 60
Time (months)
Number at risk
170 155 144 132 103 47
172 162 146 133 112 68
84 76 72 66 47 26
93 85 76 68 52 34
0 12 24 36 48 60
Time (months)
B
No RCC−type symptoms
Non−RCC related local ± systemic
RCC related local only
RCC related systemic +/− local
CSS %
100
75
50
25
0
Log−rank p−value < 0.01
0 12 24 36 48 60
Time (months)
Number at risk
182 172 160 143 111 51
181 172 156 142 116 72
94 89 81 75 55 29
123 104 92 83 65 38
0 12 24 36 48 60
Time (months)

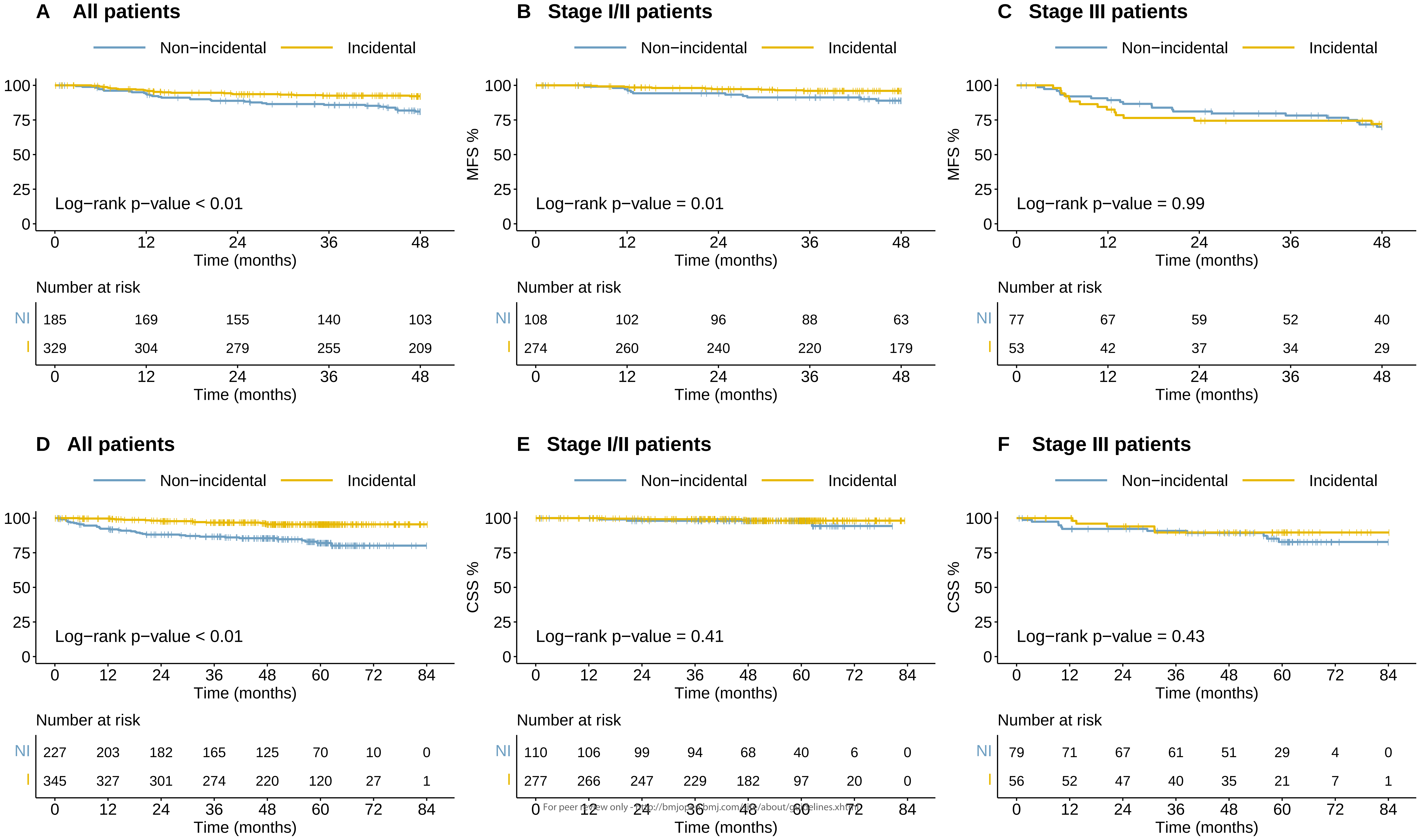
A All patients
Non−incidental
Incidental
MFS %
Log−rank p−value < 0.01
Time (months)
Number at risk
NI 185 169 155 140 103
I 329 304 279 255 209
B Stage I/II patients
Non−incidental
Incidental
MFS %
Log−rank p−value = 0.01
Time (months)
Number at risk
NI 108 102 96 88 63
I 274 260 240 220 179
C Stage III patients
Non−incidental
Incidental
MFS %
Log−rank p−value = 0.99
Time (months)
Number at risk
NI 77 67 59 52 40
I 53 42 37 34 29
D All patients
Non−incidental
Incidental
CSS %
Log−rank p−value < 0.01
Time (months)
Number at risk
NI 227 203 182 165 125 70 10 0
I 345 327 301 274 220 120 27 1
E Stage I/II patients
Non−incidental
Incidental
CSS %
Log−rank p−value = 0.41
Time (months)
Number at risk
NI 110 106 99 94 68 40 6 0
I 277 266 247 229 182 97 20 0
F Stage III patients
Non−incidental
Incidental
CSS %
Log−rank p−value = 0.43
Time (months)
Number at risk
NI 79 71 67 61 51 29 4 0
I 56 52 47 40 35 21 7 1

STROBE Statement—Checklist of items that should be included in reports of ***cohort studies***

	Item No	Recommendation	Page No
**Title and abstract**	1	(*a*) Indicate the study's design with a commonly used term in the title or the abstract<br>(*b*) Provide in the abstract an informative and balanced summary of what was done and what was found	1, 2
**Introduction**			
Background/rationale	2	Explain the scientific background and rationale for the investigation being reported	4
Objectives	3	State specific objectives, including any prespecified hypotheses	4
**Methods**			
Study design	4	Present key elements of study design early in the paper	5
Setting	5	Describe the setting, locations, and relevant dates, including periods of recruitment, exposure, follow-up, and data collection	7
Participants	6	(*a*) Give the eligibility criteria, and the sources and methods of selection of participants. Describe methods of follow-up<br>(*b*) For matched studies, give matching criteria and number of exposed and unexposed	Referenced
Variables	7	Clearly define all outcomes, exposures, predictors, potential confounders, and effect modifiers. Give diagnostic criteria, if applicable	5,6
Data sources/ measurement	8*	For each variable of interest, give sources of data and details of methods of assessment (measurement). Describe comparability of assessment methods if there is more than one group	5
Bias	9	Describe any efforts to address potential sources of bias	-
Study size	10	Explain how the study size was arrived at	7
Quantitative variables	11	Explain how quantitative variables were handled in the analyses. If applicable, describe which groupings were chosen and why	7-9
Statistical methods	12	(*a*) Describe all statistical methods, including those used to control for confounding<br>(*b*) Describe any methods used to examine subgroups and interactions<br>(*c*) Explain how missing data were addressed<br>(*d*) If applicable, explain how loss to follow-up was addressed<br>(*e*) Describe any sensitivity analyses	6 and table legends
**Results**			
Participants	13*	(a) Report numbers of individuals at each stage of study—eg numbers potentially eligible, examined for eligibility, confirmed eligible, included in the study, completing follow-up, and analysed<br>(b) Give reasons for non-participation at each stage<br>(c) Consider use of a flow diagram	7
Descriptive data	14*	(a) Give characteristics of study participants (eg demographic, clinical, social) and information on exposures and potential confounders<br>(b) Indicate number of participants with missing data for each variable of interest<br>(c) Summarise follow-up time (eg, average and total amount)	Tables P7
Outcome data	15*	Report numbers of outcome events or summary measures over time	Figures

For peer review only

Main results	16	(*a*) Give unadjusted estimates and, if applicable, confounder-adjusted estimates and their precision (eg, 95% confidence interval). Make clear which confounders were adjusted for and why they were included<br>(*b*) Report category boundaries when continuous variables were categorized<br>(*c*) If relevant, consider translating estimates of relative risk into absolute risk for a meaningful time period	NA
Other analyses	17	Report other analyses done—eg analyses of subgroups and interactions, and sensitivity analyses	-
**Discussion**			
Key results	18	Summarise key results with reference to study objectives	10-14
Limitations	19	Discuss limitations of the study, taking into account sources of potential bias or imprecision. Discuss both direction and magnitude of any potential bias	3<br>10-14
Interpretation	20	Give a cautious overall interpretation of results considering objectives, limitations, multiplicity of analyses, results from similar studies, and other relevant evidence	10-14
Generalisability	21	Discuss the generalisability (external validity) of the study results	10-14
**Other information**			
Funding	22	Give the source of funding and the role of the funders for the present study and, if applicable, for the original study on which the present article is based	14

*Give information separately for exposed and unexposed groups.

Note: An Explanation and Elaboration article discusses each checklist item and gives methodological background and published examples of transparent reporting. The STROBE checklist is best used in conjunction with this article (freely available on the Web sites of PLoS Medicine at http://www.plosmedicine.org/, Annals of Internal Medicine at http://www.annals.org/, and Epidemiology at http://www.epidem.com/). Information on the STROBE Initiative is available at http://www.strobe-statement.org.

For peer review only

For peer review only - http://bmjopen.bmj.com/site/about/guidelines.xhtml

BMJ Open

The challenge of early renal cancer detection: symptom patterns and incidental diagnosis rate in a multicentre prospective UK cohort of patients presenting with suspected renal cancer

Journal:	*BMJ Open*
Manuscript ID	bmjopen-2019-035938.R1
Article Type:	Original research
Date Submitted by the Author:	17-Jan-2020
Complete List of Authors:	Vasudev, Naveen; University of Leeds, Leeds Institute of Medical Research at St James's<br>Wilson, Michelle; University of Leeds, Leeds Institute of Medical Research at St James's<br>Stewart, Grant; University of Cambridge; NHS Lothian<br>Adeyoju, Adebanji; Stockport NHS Foundation Trust<br>Cartledge, Jon; Saint James's University Hospital<br>Kimuli, Michael; Saint James's University Hospital<br>Datta, Shibendra; University Hospital of Wales Healthcare NHS Trust<br>Hanbury, Damian; Lister Hospital<br>Hrouda, David; Charing Cross Hospital<br>Oades, Grenville; Queen Elizabeth University Hospital<br>Patel, Poulam ; University of Nottingham, Division of Cancer and Stem Cell<br>Soomro, Naeem; Newcastle Upon Tyne Hospitals NHS Foundation Trust<br>Sullivan, Mark; Oxford University Hospitals NHS Foundation Trust<br>Webster, Jeff; Northwick Park Hospital<br>Selby, Peter; University of Leeds, Leeds Institute of Medical Research at St James's<br>Banks, Rosamonde; University of Leeds, Leeds Institute of Medical Research at St James's
**Primary Subject Heading**:	Oncology
Secondary Subject Heading:	Oncology, Public health, Health policy
Keywords:	Kidney tumours < ONCOLOGY, PUBLIC HEALTH, Health policy < HEALTH SERVICES ADMINISTRATION & MANAGEMENT

SCHOLARONE™
Manuscripts

I, the Submitting Author has the right to grant and does grant on behalf of all authors of the Work (as defined in the below author licence), an exclusive licence and/or a non-exclusive licence for contributions from authors who are: i) UK Crown employees; ii) where BMJ has agreed a CC-BY licence shall apply, and/or iii) in accordance with the terms applicable for US Federal Government officers or employees acting as part of their official duties; on a worldwide, perpetual, irrevocable, royalty-free basis to BMJ Publishing Group Ltd ("BMJ") its licensees and where the relevant Journal is co-owned by BMJ to the co-owners of the Journal, to publish the Work in this journal and any other BMJ products and to exploit all rights, as set out in our licence.

The Submitting Author accepts and understands that any supply made under these terms is made by BMJ to the Submitting Author unless you are acting as an employee on behalf of your employer or a postgraduate student of an affiliated institution which is paying any applicable article publishing charge ("APC") for Open Access articles. Where the Submitting Author wishes to make the Work available on an Open Access basis (and intends to pay the relevant APC), the terms of reuse of such Open Access shall be governed by a Creative Commons licence – details of these licences and which Creative Commons licence will apply to this Work are set out in our licence referred to above.

Other than as permitted in any relevant BMJ Author's Self Archiving Policies, I confirm this Work has not been accepted for publication elsewhere, is not being considered for publication elsewhere and does not duplicate material already published. I confirm all authors consent to publication of this Work and authorise the granting of this licence.

For peer review only

The challenge of early renal cancer detection: symptom patterns and incidental diagnosis rate in a multicentre prospective UK cohort of patients presenting with suspected renal cancer

Naveen S. Vasudev[1], Michelle Wilson[1], Grant D. Stewart[2*], Adebanji Adeyoju[3], Jon Cartledge[4], Michael Kimuli[4], Shibendra Datta[5], Damian Hanbury[6], David Hrouda[7], Grenville Oades[8], Poulam Patel[9], Naeem Soomro[10], Mark Sullivan[11], Jeff Webster[12], Peter J. Selby[1], Rosamonde E. Banks[1]

[1]Leeds Institute of Medical Research at St James's, St. James's University Hospital, Beckett Street, Leeds LS9 7TF, UK.
[2]NHS Lothian, Edinburgh, UK.
[3]Stockport NHS Foundation Trust, Stockport, UK.
[4]Department of Urology, St James's University Hospital, Beckett Street, Leeds LS9 7TF, UK.
[5]University Hospital of Wales, Cardiff Heath Park, Cardiff, Wales CF14 4XW
[6]Lister Hospital, Stevenage SG1 4AB UK
[7]Charing Cross Hospital, Imperial College Healthcare NHS Trust, London W6 8RF
[8]Queen Elizabeth University Hospital, Glasgow G51 4TF UK
[9]Division of Cancer & Stem Cells, School of Medicine, University of Nottingham, Nottingham UK
[10]Newcastle Upon Tyne Hospitals NHS Foundation Trust, Newcastle upon Tyne, UK
[11]Oxford University Hospitals NHS Foundation Trust, Oxford UK
[12]Northwick Park Hospital Watford Road Harrow, HA1 3UJ UK
*Current affiliation: Department of Surgery, University of Cambridge, Cambridge Biomedical Campus, Addenbrooke's Hospital, Cambridge, CB2 0QQ, UK

Corresponding author: Dr N Vasudev, St. James's University Hospital, Beckett Street, Leeds LS9 7TF, UK. Tel: +44 113 2067680; Fax: +44 113 2429886; e-mail: n.vasudev@leeds.ac.uk

Key words: Diagnosis; Haematuria; Incidental; Presentation; Renal cell carcinoma; Symptoms

For peer review only - http://bmjopen.bmj.com/site/about/guidelines.xhtml

Abstract

Objectives: To describe the frequency and nature of symptoms in patients presenting with suspected renal cell carcinoma (RCC) and examine their reliability in achieving early diagnosis

Design: Multicentre prospective observational cohort study

Setting and Participants: Eleven UK centres recruiting patients presenting with suspected newly diagnosed RCC. Symptoms reported by patients were recorded and reviewed. Comprehensive clinico-pathological and outcome data were also collected.

Outcomes: Type and frequency of reported symptoms. Incidental diagnosis rate. Metastasis-free and cancer-specific survival.

Results: From 706 patients recruited between 2011-2014, 608 patients with a confirmed RCC formed the primary study population. The majority (60%) of patients were diagnosed incidentally. 87% patients with stage Ia and 36% with stage III or IV disease presented incidentally. Visible haematuria was reported in 23% of patients and was commonly associated with advanced disease (49% had stage III or IV disease). Symptomatic presentation was associated with poorer outcomes, likely reflecting the presence of higher stage disease. Symptom patterns amongst the 54 patients subsequently found to have a benign renal mass were similar to those with a confirmed RCC.

Conclusions: Raising public awareness of RCC-related symptoms as a strategy to improve early detection rates is limited by the fact that related symptoms are relatively uncommon and often associated with advanced disease. Greater attention must be paid to the feasibility of screening strategies and the identification of circulating diagnostic markers.

For peer review only - http://bmjopen.bmj.com/site/about/guidelines.xhtml

Strengths and limitations of this study

- The multicentre, prospective nature of this study, amongst a contemporary cohort of UK patients, is unique and represents an important strength over previous studies
- Comprehensive linked clinico-pathological and outcome data was available for all patients
- Symptoms amongst patients subsequently found to have a benign renal mass are reported in parallel
- This was not a population-based study and our cohort represents only a small proportion of all patients diagnosed with RCC in the UK within the study period
- Patient reported symptoms were recorded following referral to secondary care and may therefore be subject to recall bias

For peer review only

Introduction

The incidence of kidney cancer in Europe is amongst the highest worldwide. In the UK, incidence rates have risen by 47% increase over the past decade, with 12,000 new cases in 2015 [1]. By 2035, it is predicted that this number will rise to over 20,000 new cases per annum and kidney cancer will come to represent the 4th commonest cancer amongst males and 9th commonest amongst females in the UK [2].

Diagnosing patients with kidney cancer can be challenging [3]. Renal cell carcinomas (RCCs), which make up the majority (85%) of kidney cancers, are characteristically insidious in onset. The once classical triad of haematuria, pain and abdominal mass is now recognised to be rare and symptoms, if present at all, can be vague, non-specific and delayed in onset. Whilst early diagnosis is recognised to be key in achieving optimal outcomes, many patients still present with advanced disease. In 2017 in England, for example, figures show that amongst patients with a recorded stage at diagnosis, 19% had stage III and 23% had stage IV disease, at the time of presentation [4].

Campaigns to raise awareness of kidney cancer amongst the public and doctors have been employed in an effort to improve early diagnosis rates [5]. Understanding how patients present may help to inform such strategies. Unlike previous studies, we prospectively collected information on symptoms reported by patients at the time of their diagnosis of suspected RCC, following recruitment to a large, contemporary, multi-institutional UK RCC biobank [6]. The aims of this sub-study were to describe symptoms reported by patients, define the current rate of incidental diagnosis and

For peer review only

look at how these factors relate to patient outcomes, with the goal of better understanding the challenges in early RCC diagnosis.

Methods

The design was a multicentre prospective observational cohort study. Patients with a renal mass suspicious of RCC, of all stages, with no prior treatment, were eligible. Full details regarding the inclusion and exclusion criteria are as previously reported [6]. Comprehensive clinical and pathological information was collected.

At the time of recruitment to the study, patients were asked about the presence and nature of symptoms leading to their diagnosis of suspected RCC, which was recorded using paper case-report forms (CRF). Specific questions relating to commonly related 'RCC-type' local symptoms (pain, haematuria, abdominal mass and/or other) and/or systemic symptoms (weight loss (any), loss of appetite, sweats, fevers, fatigue and/or other) were recorded. In addition, the investigator completing the CRF was asked to state whether the diagnosis was incidental in nature and included a subsequent free-text box requesting a description of how the patient was diagnosed. All cases were independently reviewed by two reviewers (NV and RB) to confirm or refute whether the diagnosis would be regarded as incidental or not (i.e. were any symptoms reported and, if so, would they be regarded as being related to the finding of RCC), with additional reference to individual electronic case notes where available. Reported presence of RCC-type symptoms, many of which, such as pain, are non-specific, was not always related to the finding of RCC and, where applicable therefore, considered incidental. Cases with insufficient data or where the

For peer review only - http://bmjopen.bmj.com/site/about/guidelines.xhtml

incidental nature of the diagnosis remained uncertain were not classified. Patients being investigated for asymptomatic hypertension were not classified as incidental [7].

Metastasis-free survival (MFS) was calculated for patients with localised disease, defined as the period from date of nephrectomy to date of distant recurrence. Patients without recurrence were censored at the date they were last known to be recurrence-free (for patients who died without recurrence this was date of death). Cancer-specific survival (CSS) was defined as the period from date of nephrectomy to the date of cancer-related death. Patients with a non-cancer related death were censored at their date of death and patients still alive were censored at the last date they were known to be alive. Kaplan-Meier plots were produced to visualise survival and the log-rank test was used to detect statistically significant difference between survival curves.

Patients were extensively involved in the design, delivery and evaluation of the NIHR Programme supporting this work. Patients were not directly involved in the design or evaluation of the current report.

For peer review only

For peer review only - http://bmjopen.bmj.com/site/about/guidelines.xhtml

Results

Between July 2011 and June 2014, 706 patients were recruited to the study from 11 UK centres. RCC was confirmed in 608 (86%) patients, amongst whom median follow-up was 4.8 yrs (IQR: 3.7, 5.2), and benign renal mass in 54 (7.6%) patients. The remaining 44 (6.4%) patients either did not undergo biopsy or nephrectomy or had no tumour in their biopsy cores (n=33), had another (not RCC) malignancy (n=5), or an alternative benign pathology (n=6) [6]. Amongst all patients with a confirmed RCC, 422 (69%) patients reported having RCC-type symptoms at diagnosis, of whom 221 (52%) reported symptoms that were considered related to the presence of RCC. Amongst these 221 patients, 97 (44%) had local symptoms only, 19 (8.6%) had systemic symptoms only and 105 (47.5%) reported having both local and systemic symptoms. Patient and tumour characteristics by symptom type are shown in **Table 1**.

Local RCC-related symptoms

Amongst the 202 (33%) patients reporting local RCC-related symptoms, 137 (68%) reported visible haematuria and 126 (62%) reported pain, with only 14 (7%) patients reporting an abdominal mass. Patients presenting with haematuria had a median pathological tumour size of 75mm (range 16-155) and almost half had stage III (37.2%) or IV (12.4%) disease. Only four patients (0.6%) presented with the classical triad of an abdominal mass, haematuria and local pain. The median tumour size amongst these four patients was 105 mm (range 80-154 mm) on preoperative cross-sectional imaging. No significant differences were present when considered by histological type, although the small number of patients with non-clear cell RCC limits this comparison.

For peer review only - http://bmjopen.bmj.com/site/about/guidelines.xhtml

Systemic RCC-related symptoms

Amongst those reporting systemic symptoms related to their RCC, fatigue (62%), weight loss (52%), sweats (38%) and loss of appetite (38%) were all commonly reported. Fever was relatively uncommon (10%). Patients with systemic symptoms were more likely to have grade 4 cancers and stage IV disease than those with local RCC-related symptoms only and those with symptoms unrelated to RCC ($p<0.01$) (**Table 1**).

Incidental diagnosis

Amongst the 582 patients in whom the nature of the diagnosis could be confidently classified, 351 (60%) cases of RCC were deemed to have been diagnosed incidentally. Patient and tumour characteristics by nature of diagnosis (incidental vs non-incidental) are shown in **Table 2**. No association with patient sex was found and distribution of histological subtype was similar between groups. Non-incidentally detected tumours were larger and of higher grade and stage than incidentally detected tumours ($p<0.01$). Amongst patients diagnosed with a localised pT1a tumour, the incidental diagnosis rate was 87%. Conversely, 22% of patients with stage IV disease were considered to have been diagnosed incidentally. The nature of the incidental diagnosis (e.g. during investigation for a known pre-existing condition versus investigation of unrelated symptoms) is shown in **Table 3**.

Tumour size

Pathological tumour size was available for 556 (91%) of patients. We looked at symptoms in patients presenting with tumours ≥10 cm. Amongst the 66 patients with a tumour ≥10cm, 31 (47%) reported haematuria at the time of presentation, 33 (50%)

For peer review only - http://bmjopen.bmj.com/site/about/guidelines.xhtml

reported pain, and abdominal mass was reported in four (6%) patients. Almost a quarter (16/66; 24%) of these patients were considered to have been diagnosed incidentally, with 10 (15%) reporting no symptoms, despite the presence of a large primary tumour. No effect of BMI was observed in relation to presence or absence of symptoms.

Outcomes

We looked at survival outcomes by both symptom type (no RCC-type symptoms or unrelated RCC-type symptoms vs. related RCC-type symptoms) and incidental versus non-incidental diagnosis. Patients diagnosed with no RCC-type symptoms and those reporting unrelated RCC-type symptoms had a significantly improved MFS and CSS compared to patients with related RCC-type symptoms. Furthermore, patients with systemic RCC-related symptoms had poorer outcomes than those with local RCC symptoms only (**Figure 1 A and B**). Overall, patients with an incidental diagnosis of RCC had improved MFS and CSS in comparison to those diagnosed non-incidentally, although it is important to note that these effects were lost when controlled for stage of disease (**Figure 2**).

Patients presenting with benign renal masses

In total, 54 (7.6%) patients in our cohort were found to have a benign renal mass, composed of oncocytoma (n=29), angiomyolipoma (n=8) and other lesions (n=17) (**Table 4**). The incidental diagnosis rate was 56% amongst the 52 evaluable patients. Haematuria and pain were reported in 57% and 52% of patients diagnosed non-incidentally. The majority (65%) reported symptoms, of whom 57% had local symptoms only, 17% had systemic symptoms only and 26% reported both local and systemic symptoms.

For peer review only - http://bmjopen.bmj.com/site/about/guidelines.xhtml

Discussion

Early detection is widely held to be a key strategy towards improving outcomes in patients with RCC [8]. As in most solid cancers, disease stage and survival are closely linked, with 3-year CSS rates in our cohort for example, of 99% and 47% for stage I and stage IV cancers, respectively (data not shown). Symptoms of kidney cancer such as visible haematuria and flank pain are well documented and NHS initiatives such as 'be clear on cancer: blood in your pee' campaign have been aimed at prompting the public to seek early medical attention [5]. Nevertheless, many patients still present with overt or micro-metastatic disease. Understanding the type and frequency of symptoms patients with newly diagnosed RCC report is critical in beginning to address this issue and understand whether simply raising awareness amongst doctors and the public is sufficient or other strategies are needed.

Our study highlights the significant challenges in diagnosing patients with kidney cancer. Almost a third of patients in our cohort were symptomless at the time of diagnosis, amongst whom nearly a quarter (24%) had stage III or IV disease. Visible haematuria, a hallmark symptom of this disease, was recorded in just 23% of patients overall. Even amongst patients with large (≥10cm) tumours, less than half (47%) reported haematuria as a symptom. Prior reports using UK general practice database records have suggested rates of haematuria as low as 18% in patients presenting with kidney cancer, compounded by the low positive predictive value (PPV) (1%) of this symptom for RCC amongst those ≥60 yr old [9]. Furthermore, symptom patterns do not appear to reliably distinguish patients with benign renal masses from those with RCC.

For peer review only - http://bmjopen.bmj.com/site/about/guidelines.xhtml

Many studies have attempted to document the incidental diagnosis rate for renal cancer. These previous studies have all been retrospective in nature, typically derived from patients at a single centre, with widely varying rates of incidental diagnosis, from 15% to 61%, in a less contemporaneous setting (broadly spanning 1970-2000) [10-14]. A more recent, global, study, involving 4288 patients presenting with RCC between 2010-2012, reported an incidental diagnosis rate of 67% [15]. However, no detail regarding how this was derived, or the nature and characteristics of those diagnosed incidentally were presented in this study. We carefully reviewed the presenting symptoms and history for each patient in our study, performed independently by two of the authors, to determine as accurately as possible whether the diagnosis would be deemed incidental or not. Pain, for example, was a commonly reported symptom not necessarily attributable to the diagnosis of RCC, for example when located in an anatomically distinct site. We believe our figure of 60%, amongst a contemporary set of patients (2011-2014), provides a true reflection of the current incidental diagnosis rate of RCC in the UK, and supports the general rise in the incidental detection of kidney cancer that has been reported over time.

Our data shows that the majority (60%) of patients with RCC in the UK are being diagnosed incidentally, with almost three-quarters of these (74%) during investigation of symptoms unrelated to RCC. By contrast, a Norwegian study of 413 patients diagnosed with RCC between 1997-2010 reported a 53% incidental diagnosis rate, detected in 63% of these patients during follow-up for a pre-existing condition [16]. The reason for this difference is not certain but may reflect the different time periods under study, given the more liberal use of cross-sectional imaging over time [17]. Consistent with other studies, patients with an incidentally detected RCC

For peer review only - http://bmjopen.bmj.com/site/about/guidelines.xhtml

tended to have smaller, lower stage and grade tumours than those presenting with related symptoms, but, nevertheless, almost one in five of patients identified incidentally had stage III/IV disease at diagnosis. Whether patients who are diagnosed incidentally have better outcomes and potentially, therefore, different tumour biology, than those presenting with symptoms has been a matter of debate in the literature [10,18-20]. We did not find any difference in MFS or CSS between these two groups when matched for stage of disease, suggesting that incidental detection of advanced stage disease is not advantageous in terms of outcome.

Diagnosing kidney cancer early is therefore a significant public health challenge. Data from the 2010 National Cancer Patient Experience Survey in England report that almost 30% of 564 patients with renal cancer saw their general practitioner three or more times before hospital referral [21]. Furthermore, results from the charity Kidney Cancer UK (KCUK) 2018 patient survey showed that 22% of the 153 responders who presented to their GP or an A+E department waited more than 3 months for a diagnosis [22]. The results of the KCUK survey (n=175 in total) extend further, with 51% of patients reporting their cancer being detected incidentally during imaging for an unrelated reason, and less than one third (31%) having symptoms due to RCC, reflecting the findings from our own, much larger, study.

How then do we improve rates of early diagnosis in kidney cancer? Raising awareness amongst the public to present early to their doctor, even with vague symptoms, may seem logical, as well as increasing awareness with primary care teams. But many patients remain asymptomatic until they have advanced stage disease, and the PPVs for symptoms other than haematuria, such as pain and

For peer review only - http://bmjopen.bmj.com/site/about/guidelines.xhtml
For peer review only

fatigue, are even lower than 1% [9], placing an impossible demand on general practitioners, who are required to act as gatekeepers to secondary care. Five-year survival rates for kidney cancer in the UK lag behind the European average which may be related to differences in stage at diagnosis [23]. Greater availability of point-of-care ultrasound may make a significant impact but its use varies widely across Europe and has not been widely adopted in the UK, with potential barriers in terms of time and training [24].

Interest in exploring the potential for kidney cancer screening is growing [8,25], particularly given the significant predicted rise in incidence [2]. The potential cost-effectiveness of performing a single, renal focused, USS amongst asymptomatic 60-year-old men has recently been reported [26]. However numerous uncertainties still exist, in terms of who to screen, with what modality, as well as unknowns in terms of associated harms versus benefit [27]. This is an area that clearly warrants further research. The identification of robust diagnostic biomarkers either in the serum or urine of patients that could be used to easily rule in or out the presence of RCC is another priority area for study [28], with recent promising reports in the literature [29], although still requiring significant further validation and improved performance.

The strengths of this study include its prospective multicentre design, amongst a contemporary cohort of patients with robust linked clinicopathological and outcome data. The eligibility criteria for the study were broad and we believe our patient cohort to be representative. Nevertheless, we acknowledge that our cohort size reflects only a small proportion (less than 10%) of all patients diagnosed with RCC in the UK during the study period. A further limitation is the fact that patient-reported

For peer review only - http://bmjopen.bmj.com/site/about/guidelines.xhtml

symptoms were recorded following referral to secondary care and there may therefore be some element of recall bias.

In summary, this study draws attention to the fact that reliance on symptoms for the early detection of kidney cancer is not robust. Our data suggest that improving public and professional awareness will have only a limited impact, and innovative biomarkers for this purpose remain to be identified. We suggest it is time to re-examine the case for screening looking at opportunities to link RCC screening into other programmes such as low dose CT scans for lung cancer health checks or ultrasound-based screening for abdominal aortic aneurysms.

Contributors: Funding: PJS and REB; Study design and concept: PJS, REB, NSV; Patient recruitment and data collection: AA, JC, MK, SD, DHa, DHr, GO, PP, NS, GDS, MS, JW, NSV, REB; Data analysis: MW, GDS, NSV, REB; Manuscript preparation: NSV, MW, GDS, PJS, REB. All authors participated in the interpretation of results, critically revised the paper and approved the final version to be published.

Ethics approval and consent to participate: The study was approved by the Leeds East Research Ethics Committee (ethical approval 10/H1306/6). Patients were recruited to the study with their written informed consent

Funding: This project was funded by the National Institute for Health Research Programme Grants for Applied Research programme (project number NIHR PGfAR RP-PG-0707-10101) and has been published in full in Programme Grants for Applied Research Volume: 6, Issue: 3. Further information is available at: https://www.journalslibrary.nihr.ac.uk/pgfar/pgfar06030/#/abstract. Additional support was provided by the NIHR Leeds Clinical Research Facility. The views and opinions expressed by the authors in this publication are those of the authors and do not necessarily reflect those of the NHS, the NIHR or the Department of Health.

Disclaimer: The funder had no role in study design, data collection, data analysis or manuscript preparation

Conflicts of interest: N.S.V. discloses paid consultancy work and/or speaker honoraria for EUSA pharma, Bristol Myers Squibb, Pfizer, Merck and Novartis. G.D.S. discloses paid consultancy work for Pfizer, EUSA Pharma, Merck, and CMR Surgical and an advisory board role for the latter. The other authors declare no conflicts of interest.

For peer review only - http://bmjopen.bmj.com/site/about/guidelines.xhtml
For peer review only

Acknowledgements: We are grateful to the patients, the staff at participating centres who participated in patient recruitment and data collection, staff within the Leeds Biobanking and Sample Processing Lab and staff at the Leeds Clinical Trials Research Unit.

Patient consent for publication: Not required

Data availability statement: All data relevant to the study are included in the article

For peer review only

References

1. Cancer Research UK. https://www.cancerresearchuk.org/health-professional/cancer-statistics-for-the-uk. Accessed August 2019.
2. Smittenaar CR, Petersen KA, Stewart K, Moitt N. Cancer incidence and mortality projections in the UK until 2035. *Br J Cancer* 2016:**115**:1147-55
3. Zhou Y, van Melle M, Singh H, Hamilton W, Lyratzopoulos G, Walter FM. Quality of the diagnostic process in patients presenting with symptoms suggestive of bladder or kidney cancer: a systematic review. *BMJ Open* 2019:**9**:e029143
4. http://ncin.org.uk/publications/survival_by_stage. Accessed August 2019
5. https://www.nhs.uk/be-clear-on-cancer/symptoms/blood-in-pee. Accessed August 2019
6. Vasudev NS, Hutchinson M, Trainor S *et al.* UK multicentre prospective evaluation of the Leibovich score in localised renal cell carcinoma: performance has altered over time. *Urology* 2019. In Press
7. Chow WH, Gridley G, Fraumeni JF, Jr., Jarvholm B. Obesity, hypertension, and the risk of kidney cancer in men. *N Engl J Med* 2000:**343**:1305-11
8. Motzer RJ. Perspective: What next for treatment? *Nature* 2016:**537**:S111
9. Shephard E, Neal R, Rose P, Walter F, Hamilton WT. Clinical features of kidney cancer in primary care: a case-control study using primary care records. *Br J Gen Pract* 2013:**63**:e250-5
10. Tsui KH, Shvarts O, Smith RB, Figlin R, de Kernion JB, Belldegrun A. Renal cell carcinoma: prognostic significance of incidentally detected tumors. *J Urol* 2000:**163**:426-30
11. Lee CT, Katz J, Fearn PA, Russo P. Mode of presentation of renal cell carcinoma provides prognostic information. *Urol Oncol* 2002:**7**:135-40
12. Ficarra V, Prayer-Galetti T, Novella G *et al.* Incidental detection beyond pathological factors as prognostic predictor of renal cell carcinoma. *Eur Urol* 2003:**43**:663-9
13. Luciani LG, Cestari R, Tallarigo C. Incidental renal cell carcinoma-age and stage characterization and clinical implications: study of 1092 patients (1982-1997). *Urology* 2000:**56**:58-62

For peer review only - http://bmjopen.bmj.com/site/about/guidelines.xhtml

14. Jayson M, Sanders H. Increased incidence of serendipitously discovered renal cell carcinoma. *Urology* 1998:**51**:203-5
15. Laguna MP, Algaba F, Cadeddu J *et al.* Current patterns of presentation and treatment of renal masses: a clinical research office of the endourological society prospective study. *J Endourol* 2014:**28**:861-70
16. Sand KE, Hjelle KM, Rogde AJ, Gudbrandsdottir G, Bostad L, Beisland C. Incidentally detected renal cell carcinomas are highly associated with comorbidity and mortality unrelated to renal cell carcinoma. *Scand J Urol* 2013:**47**:462-71
17. https://www.gov.uk/government/publications/review-of-radiation-dose-issues-from-the-use-of-ct-in-the-uk. Accessed August 2019
18. Patard JJ, Rodriguez A, Rioux-Leclercq N, Guille F, Lobel B. Prognostic significance of the mode of detection in renal tumours. *BJU Int* 2002:**90**:358-63
19. Palsdottir HB, Hardarson S, Petursdottir V *et al.* Incidental detection of renal cell carcinoma is an independent prognostic marker: results of a long-term, whole population study. *J Urol* 2012:**187**:48-53
20. Gudbjartsson T, Thoroddsen A, Petursdottir V, Hardarson S, Magnusson J, Einarsson GV. Effect of incidental detection for survival of patients with renal cell carcinoma: results of population-based study of 701 patients. *Urology* 2005:**66**:1186-91
21. Lyratzopoulos G, Neal RD, Barbiere JM, Rubin GP, Abel GA. Variation in number of general practitioner consultations before hospital referral for cancer: findings from the 2010 National Cancer Patient Experience Survey in England. *Lancet Oncol* 2012:**13**:353-65
22. https://www.kcuk.org.uk/patient-survey-2018/. Accessed August 2019
23. De Angelis R, Sant M, Coleman MP *et al.* Cancer survival in Europe 1999-2007 by country and age: results of EUROCARE--5-a population-based study. *Lancet Oncol* 2014:**15**:23-34
24. Mengel-Jorgensen T, Jensen MB. Variation in the use of point-of-care ultrasound in general practice in various European countries. Results of a survey among experts. *Eur J Gen Pract* 2016:**22**:274-7
25. Shea MW. A proposal for a targeted screening program for renal cancer. *Front Oncol* 2013:**3**:207
26. Rossi SH, Klatte T, Usher-Smith JA *et al.* A Decision Analysis Evaluating Screening for Kidney Cancer Using Focused Renal Ultrasound. *Eur Urol Focus* 2019
27. Rossi SH, Klatte T, Usher-Smith J, Stewart GD. Epidemiology and screening for renal cancer. *World J Urol* 2018:**36**:1341-53
28. Capitanio U, Bensalah K, Bex A *et al.* Epidemiology of Renal Cell Carcinoma. *Eur Urol* 2019:**75**:74-84
29. Morrissey JJ, Mellnick VM, Luo J *et al.* Evaluation of Urine Aquaporin-1 and Perilipin-2 Concentrations as Biomarkers to Screen for Renal Cell Carcinoma: A Prospective Cohort Study. *JAMA Oncol* 2015:**1**:204-12

For peer review only

For peer review only - http://bmjopen.bmj.com/site/about/guidelines.xhtml

Figure Legends

Figure 1. Kaplan Meier survival curves by symptom type. Survival outcomes (A. MFS; B. CSS) in patients with no RCC-type symptoms, unrelated RCC-type symptoms, local RCC-related symptoms and those with systemic (+/- local) RCC-related symptoms

Figure 2. Kaplan Meier survival curves by incidental vs. non-incidental diagnosis for all patients, stage I/II or stage III RCC. A-C: MFS; D-F: CSS

For peer review only

For peer review only - http://bmjopen.bmj.com/site/about/guidelines.xhtml

Table 1. Patient and tumour characteristics by symptom type
For continuous variables, figures in table represent median (range) with corresponding p-value from the Kruskal-Wallis test and for categorical variables, figures in table represent n (%) with corresponding p-value from chi-squared test.

		RCC-type symptoms reported (n=422)**			
**Characteristic**	**No RCC-type symptoms (n=186)**	**Not RCC related (n=183)**	**RCC-related local symptoms only (n=97)**	**RCC-related systemic symptoms (+/- local) (n=124)**	**p-value**
**Age (years)**	65 (31-86)	63 (29-90)	63 (38-84)	62 (33-92)	0.31
**Gender**					
**Female**	67 (32.7)	62 (30.2)	21 (10.2)	55 (26.8)	
**Male**	119 (30.9)	121 (31.4)	76 (19.7)	69 (17.9)	0.01
**BMI**	28.5 (15.6-74.4)	27 (18.1-56.5)	28.8 (17.3-67.2)	27.5 (16-54.5)	0.01
**Tumour size (mm)**	44 (14-180)	43 (11-170)	74 (13-155)	75 (20-240)	<0.01
**pT**					
**1a**	83 (42.6)	88 (45.1)	16 (8.2)	8 (4.1)	
**1b**	46 (34.3)	42 (31.3)	19 (14.2)	27 (20.1)	
**2**	15 (19.7)	18 (23.7)	19 (25)	24 (31.6)	
**3**	38 (22.6)	33 (19.6)	42 (25)	55 (32.7)	
**4**	0 (0)	0 (0)	1 (25)	3 (75)	
**X**	1 (50)	1 (50)	0 (0)	0 (0)	
**Missing**	1 (100)	0 (0)	0 (0)	0 (0)	
**NA**	2 (20)	1 (10)	0 (0)	7 (70)	<0.01
**Grade**					
**1**	4 (40)	0 (0)	4 (40)	2 (20)	
**2**	55 (34.8)	50 (31.6)	25 (15.8)	28 (17.7)	
**3**	88 (32.2)	94 (34.4)	47 (17.2)	44 (16.1)	
**4**	13 (14.9)	13 (14.9)	19 (21.8)	42 (48.3)	
**Missing**	9 (39.1)	9 (39.1)	1 (4.3)	4 (17.4)	
**NA**	17 (43.6)	17 (43.6)	1 (2.6)	4 (10.3)	<0.01
**Stage**					
**I**	130 (39.8)	129 (39.4)	34 (10.4)	34 (10.4)	
**II**	12 (17.4)	17 (24.6)	18 (26.1)	22 (31.9)	
**III**	34 (24.5)	29 (20.9)	37 (26.6)	39 (28.1)	
**IV**	10 (18.9)	6 (11.3)	8 (15.1)	29 (54.7)	
**Missing**	0 (0)	2 (100)	0 (0)	0 (0)	<0.01
**Tumour subtype**					
**Clear Cell**	147 (31.7)	137 (29.6)	83 (17.9)	96 (20.7)	
**Papillary**	16 (27.1)	23 (39)	7 (11.9)	13 (22)	
**Chromophobe**	15 (32.6)	15 (30.4)	7 (15.2)	10 (21.7)	
**Unclassified**	7 (38.9)	6 (33.3)	0 (0)	5 (27.8)	
**Other**	1 (33)	2 (67)	0 (0)	0 (0)	0.81

*NA=not applicable - patients underwent biopsy only or tumour ablation
**18 patients reported symptoms but their relationship to RCC could not be determined

For peer review only - http://bmjopen.bmj.com/site/about/guidelines.xhtml

Table 2. Patient and tumour characteristics by diagnosis type
For continuous variables, figures in table represent median (range) with corresponding p-value from the Wilcoxon rank-sum test and for categorical variables, figures in table represent n (%) with corresponding p-value from chi-squared test.

Characteristic	Non-incidental (n=231)	Incidental (n=351)	p-value
Age (years)	62 (33-92)	65 (29-90)	0.04
Gender			
Female	77 (38.3)	124 (61.7)	
Male	154 (40.4)	227 (59.6)	0.69
BMI	28.3 (15.6-67.2)	27.8 (17.2-57.7)	0.38
Tumour size (path) (mm)	75 (13-240)	42 (11-170)	<0.01
Tumour size (CT) (mm)	80 (16-250)	44 (10-170)	<0.01
pT			
1a	25 (12.8)	170 (87.2)	
1b	48 (37.2)	81 (62.8)	
2	46 (60.5)	30 (39.5)	
3	101 (61.2)	64 (38.8)	
4	4 (100)	0 (0)	
X	0 (0)	2 (100)	
Missing	0 (0)	1 (100)	
NA*	7 (70)	3 (30)	<0.01
Grade			
1	6 (66.7)	3 (33.3)	
2	56 (35.9)	100 (64.1)	
3	93 (34.6)	176 (65.4)	
4	65 (75.6)	21 (24.4)	
Missing	6 (26.1)	17 (73.9)	
NA*	5 (12.8)	34 (87.2)	<0.01
Stage			
I	70 (21.6)	254 (78.4)	
II	42 (60.9)	27 (39.1)	
III	80 (58.4)	57 (41.6)	
IV	39 (78)	11 (22)	
Missing	0 (0)	2 (100)	<0.01
Tumour subtype			
Clear Cell	186 (40.9)	269 (59.1)	
Papillary	21 (35.6)	38 (64.4)	
Chromophobe	19 (41.3)	28 (58.7)	
Unclassified	5 (27.8)	13 (72.2)	
Other	0 (0)	3 (100)	0.62

*NA=not applicable, patients underwent biopsy only or tumour ablation

For peer review only - http://bmjopen.bmj.com/site/about/guidelines.xhtml

Table 3. Nature of incidental diagnosis

Type of incidental diagnosis	n (%)
**Investigation for pre-existing condition**	65 (18)
Another malignancy	34 (53)
Diabetes Mellitus	7 (11)
Hepatobiliary[a]	5 (8)
AAA screening / Post-aortic repair	3 (5)
Other[b]	16 (23)
**Investigation for signs or symptoms unrelated to RCC**	258 (74)
Gastrointestinal[c]	86 (33)
Urinary tract[d]	49 (19)
Hepatobiliary[e]	27 (10)
Respiratory[f]	20 (8)
Musculoskeletal[g]	16 (6)
Cardiovascular[h]	11 (4)
Trauma	7 (3)
Gynaecological	6 (3)
Anaemia	4 (2)
Miscellaneous[i]	32 (12)
**Routine health check**[k]	16 (5)
**Not known**[l]	12 (3)

AAA, abdominal aortic aneurysm; [a]cirrhosis, primary biliary cirrhosis, sclerosing cholangitis; [b]includes Addison's disease, chronic renal failure, crohn's disease, coeliac disease, ovarian cyst, renal stones, IgA nephropathy, Wegener's granulomatosis, polymyalgia rheumatica, ovarian cyst; [c]altered bowel habit, GI bleed, bloating/distension, abdominal pain, reflux; [d]urinary retention, prostatic symptoms, high PSA, urosepsis, renal colic, impaired renal function; [e]biliary colic, deranged liver function tests, jaundice, pancreatitis, cholecystitis; [f]shortness of breath, cough, haemoptysis, pneumonia; [g]back pain, leg pain, joint pain; [h]chest pain, myocardial infarction, claudication, endocarditis; [i]includes dizziness, syncope, elevated blood test values, ankle swelling; [k]Initial investigations were urine dip (6), USS (5), CT scan (2), blood tests (2), CXR (1); [l]insufficient information to classify

For peer review only - http://bmjopen.bmj.com/site/about/guidelines.xhtml

Table 4. Characteristics and symptoms associated with benign renal masses

Characteristic	All (n=54)	Oncocytoma (n=29)	AML (n=8)	Other* (n=17)
**Age (years)**	65 (32-86)	66 (42-86)	63 (59-68)	61 (32-78)
**Gender**				
**Female**	29 (53.7)	12 (41.4)	5 (62.5)	12 (70.6)
**Male**	25 (46.3)	17 (58.6)	3 (37.5)	5 (29.4)
**BMI**	27.6 (18.7-45.8)	27.8 (19.4-39.6)	28 (22-38.8)	26.4 (18.7-45.8)
**CT size (cm)**				
**≤4**	22 (44.9)	14 (50)	3 (50)	5 (33.3)
**4< - ≤7**	18 (36.7)	11 (39.3)	2 (33.3)	5 (33.3)
**7< - ≤10**	6 (12.2)	1 (3.6)	1 (16.7)	4 (26.7)
**>10**	3 (6.1)	2 (7.1)	0 (0)	1 (6.7)
**NA**	5 (-)	1 (-)	2 (-)	2 (-)
**RCC-type symptoms**				
**No**	19 (35.2)	10 (34.5)	4 (50)	5 (29.4)
**Yes**	35 (64.8)	19 (65.5)	4 (50)	12 (70.6)
**Local symptoms**				
**No**	6 (17.1)	3 (15.8)	1 (25)	2 (16.7)
**Yes**	29 (82.9)	16 (84.2)	3 (75)	10 (83.3)
**Systemic symptoms**				
**No**	20 (57.1)	12 (63.2)	1 (25)	7 (58.3)
**Yes**	15 (42.9)	7 (36.8)	3 (75)	5 (41.7)
**Incidental diagnosis**				
**No**	23 (42.5)	13 (44.8)	2 (25)	8 (47)
**Yes**	29 (54)	15 (51.7)	6 (75)	8 (47)
**Not known**	2 (3.5)	1 (3.5)	0 (0)	1 (6)

AML – angiomyolipoma *consists of cystic nephroma (4), benign cyst (3), metanephric adenoma (2), mixed epithelial stromal tumour (2), haemangioblastoma (1), leiomyomata (1), multilocular cyst (1), myxoid mesenchymal tumour (1), Rosai Dorfman disease (1), solitary fibrous tumour (1)

For peer review only

For peer review only - http://bmjopen.bmj.com/site/about/guidelines.xhtml

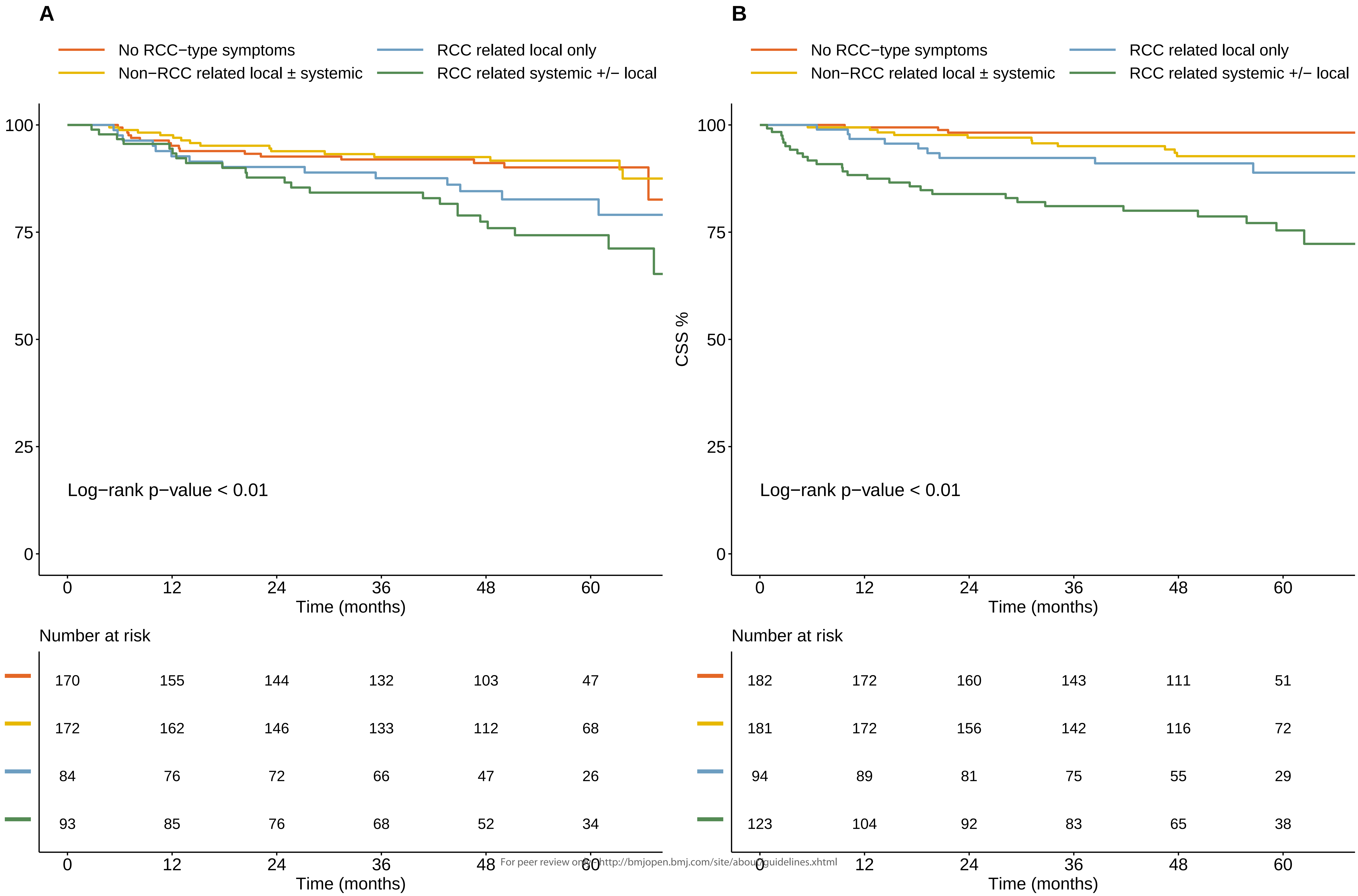
A
No RCC−type symptoms
Non−RCC related local ± systemic
RCC related local only
RCC related systemic +/− local
MFS %
100
75
50
25
0
Log−rank p−value < 0.01
0 12 24 36 48 60
Time (months)
Number at risk
170 155 144 132 103 47
172 162 146 133 112 68
84 76 72 66 47 26
93 85 76 68 52 34
0 12 24 36 48 60
Time (months)
B
No RCC−type symptoms
Non−RCC related local ± systemic
RCC related local only
RCC related systemic +/− local
CSS %
100
75
50
25
0
Log−rank p−value < 0.01
0 12 24 36 48 60
Time (months)
Number at risk
182 172 160 143 111 51
181 172 156 142 116 72
94 89 81 75 55 29
123 104 92 83 65 38
0 12 24 36 48 60
Time (months)

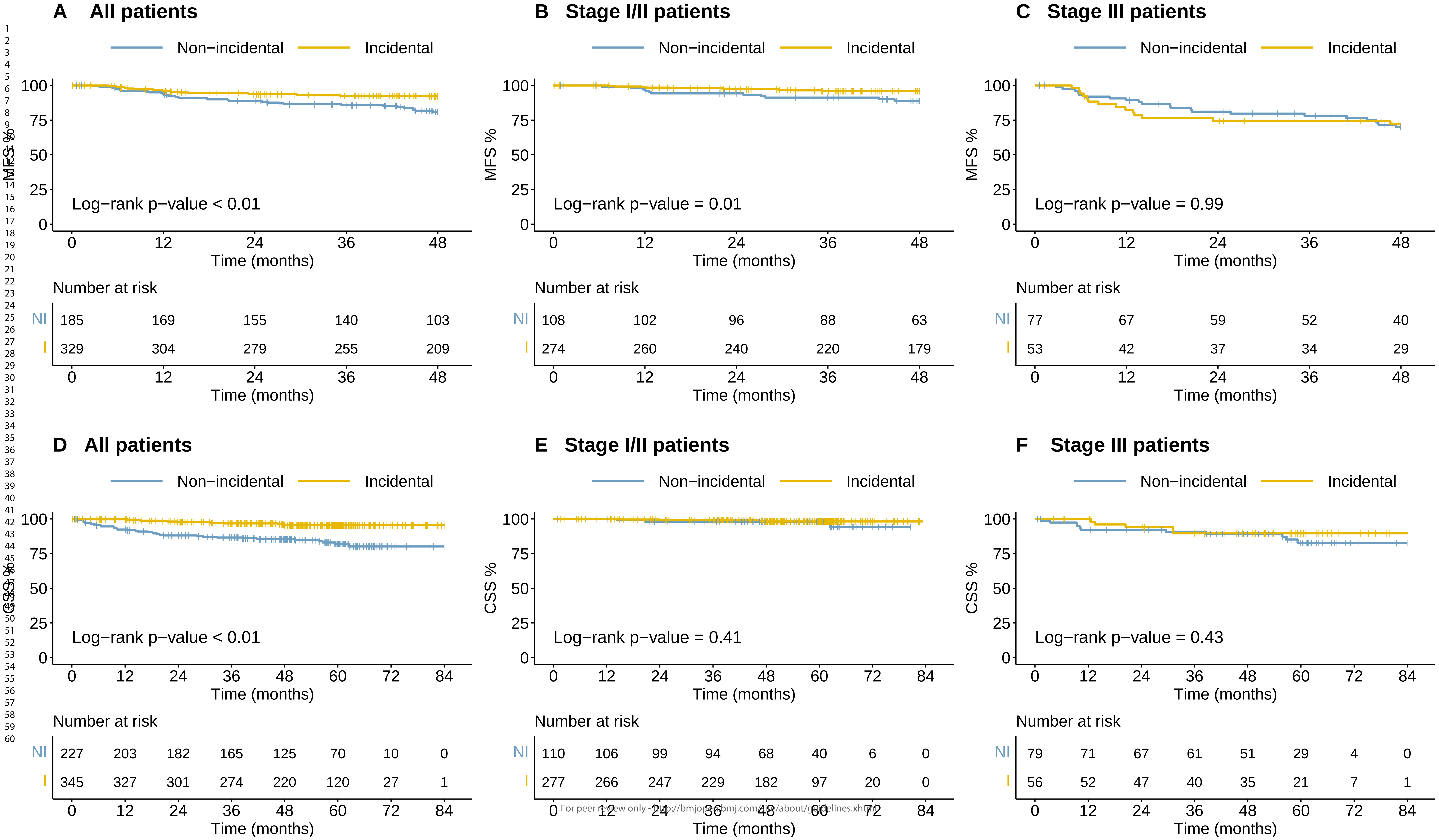

For peer review only - http://bmjopen.bmj.com/site/about/guidelines.xhtml

STROBE Statement—Checklist of items that should be included in reports of ***cohort studies***

	Item No	Recommendation	Page No
**Title and abstract**	1	(*a*) Indicate the study's design with a commonly used term in the title or the abstract<br>(*b*) Provide in the abstract an informative and balanced summary of what was done and what was found	1, 2
**Introduction**			
Background/rationale	2	Explain the scientific background and rationale for the investigation being reported	4
Objectives	3	State specific objectives, including any prespecified hypotheses	4
**Methods**			
Study design	4	Present key elements of study design early in the paper	5
Setting	5	Describe the setting, locations, and relevant dates, including periods of recruitment, exposure, follow-up, and data collection	7
Participants	6	(*a*) Give the eligibility criteria, and the sources and methods of selection of participants. Describe methods of follow-up<br>(*b*) For matched studies, give matching criteria and number of exposed and unexposed	Referenced
Variables	7	Clearly define all outcomes, exposures, predictors, potential confounders, and effect modifiers. Give diagnostic criteria, if applicable	5,6
Data sources/ measurement	8*	For each variable of interest, give sources of data and details of methods of assessment (measurement). Describe comparability of assessment methods if there is more than one group	5
Bias	9	Describe any efforts to address potential sources of bias	-
Study size	10	Explain how the study size was arrived at	7
Quantitative variables	11	Explain how quantitative variables were handled in the analyses. If applicable, describe which groupings were chosen and why	7-9
Statistical methods	12	(*a*) Describe all statistical methods, including those used to control for confounding<br>(*b*) Describe any methods used to examine subgroups and interactions<br>(*c*) Explain how missing data were addressed<br>(*d*) If applicable, explain how loss to follow-up was addressed<br>(*e*) Describe any sensitivity analyses	6 and table legends
**Results**			
Participants	13*	(a) Report numbers of individuals at each stage of study—eg numbers potentially eligible, examined for eligibility, confirmed eligible, included in the study, completing follow-up, and analysed<br>(b) Give reasons for non-participation at each stage<br>(c) Consider use of a flow diagram	7
Descriptive data	14*	(a) Give characteristics of study participants (eg demographic, clinical, social) and information on exposures and potential confounders<br>(b) Indicate number of participants with missing data for each variable of interest<br>(c) Summarise follow-up time (eg, average and total amount)	Tables P7
Outcome data	15*	Report numbers of outcome events or summary measures over time	Figures

For peer review only - http://bmjopen.bmj.com/site/about/guidelines.xhtml

Main results	16	(*a*) Give unadjusted estimates and, if applicable, confounder-adjusted estimates and their precision (eg, 95% confidence interval). Make clear which confounders were adjusted for and why they were included<br>(*b*) Report category boundaries when continuous variables were categorized<br>(*c*) If relevant, consider translating estimates of relative risk into absolute risk for a meaningful time period	NA
Other analyses	17	Report other analyses done—eg analyses of subgroups and interactions, and sensitivity analyses	-
**Discussion**			
Key results	18	Summarise key results with reference to study objectives	10-14
Limitations	19	Discuss limitations of the study, taking into account sources of potential bias or imprecision. Discuss both direction and magnitude of any potential bias	3<br>10-14
Interpretation	20	Give a cautious overall interpretation of results considering objectives, limitations, multiplicity of analyses, results from similar studies, and other relevant evidence	10-14
Generalisability	21	Discuss the generalisability (external validity) of the study results	10-14
**Other information**			
Funding	22	Give the source of funding and the role of the funders for the present study and, if applicable, for the original study on which the present article is based	14

*Give information separately for exposed and unexposed groups.

Note: An Explanation and Elaboration article discusses each checklist item and gives methodological background and published examples of transparent reporting. The STROBE checklist is best used in conjunction with this article (freely available on the Web sites of PLoS Medicine at http://www.plosmedicine.org/, Annals of Internal Medicine at http://www.annals.org/, and Epidemiology at http://www.epidem.com/). Information on the STROBE Initiative is available at http://www.strobe-statement.org.

For peer review only

For peer review only - http://bmjopen.bmj.com/site/about/guidelines.xhtml

BMJ Open

The challenge of early renal cancer detection: symptom patterns and incidental diagnosis rate in a multicentre prospective UK cohort of patients presenting with suspected renal cancer

Journal:	*BMJ Open*
Manuscript ID	bmjopen-2019-035938.R2
Article Type:	Original research
Date Submitted by the Author:	17-Feb-2020
Complete List of Authors:	Vasudev, Naveen; University of Leeds, Leeds Institute of Medical Research at St James's<br>Wilson, Michelle; University of Leeds, Leeds Institute of Medical Research at St James's<br>Stewart, Grant; University of Cambridge; NHS Lothian<br>Adeyoju, Adebanji; Stockport NHS Foundation Trust<br>Cartledge, Jon; Saint James's University Hospital<br>Kimuli, Michael; Saint James's University Hospital<br>Datta, Shibendra; University Hospital of Wales Healthcare NHS Trust<br>Hanbury, Damian; Lister Hospital<br>Hrouda, David; Charing Cross Hospital<br>Oades, Grenville; Queen Elizabeth University Hospital<br>Patel, Poulam ; University of Nottingham, Division of Cancer and Stem Cell<br>Soomro, Naeem; Newcastle Upon Tyne Hospitals NHS Foundation Trust<br>Sullivan, Mark; Oxford University Hospitals NHS Foundation Trust<br>Webster, Jeff; Northwick Park Hospital<br>Selby, Peter; University of Leeds, Leeds Institute of Medical Research at St James's<br>Banks, Rosamonde; University of Leeds, Leeds Institute of Medical Research at St James's
**Primary Subject Heading**:	Oncology
Secondary Subject Heading:	Oncology, Public health, Health policy
Keywords:	Kidney tumours < ONCOLOGY, PUBLIC HEALTH, Health policy < HEALTH SERVICES ADMINISTRATION & MANAGEMENT

SCHOLARONE™
Manuscripts

I, the Submitting Author has the right to grant and does grant on behalf of all authors of the Work (as defined in the below author licence), an exclusive licence and/or a non-exclusive licence for contributions from authors who are: i) UK Crown employees; ii) where BMJ has agreed a CC-BY licence shall apply, and/or iii) in accordance with the terms applicable for US Federal Government officers or employees acting as part of their official duties; on a worldwide, perpetual, irrevocable, royalty-free basis to BMJ Publishing Group Ltd ("BMJ") its licensees and where the relevant Journal is co-owned by BMJ to the co-owners of the Journal, to publish the Work in this journal and any other BMJ products and to exploit all rights, as set out in our licence.

The Submitting Author accepts and understands that any supply made under these terms is made by BMJ to the Submitting Author unless you are acting as an employee on behalf of your employer or a postgraduate student of an affiliated institution which is paying any applicable article publishing charge ("APC") for Open Access articles. Where the Submitting Author wishes to make the Work available on an Open Access basis (and intends to pay the relevant APC), the terms of reuse of such Open Access shall be governed by a Creative Commons licence – details of these licences and which Creative Commons licence will apply to this Work are set out in our licence referred to above.

Other than as permitted in any relevant BMJ Author's Self Archiving Policies, I confirm this Work has not been accepted for publication elsewhere, is not being considered for publication elsewhere and does not duplicate material already published. I confirm all authors consent to publication of this Work and authorise the granting of this licence.

For peer review only

The challenge of early renal cancer detection: symptom patterns and incidental diagnosis rate in a multicentre prospective UK cohort of patients presenting with suspected renal cancer

Naveen S. Vasudev[1], Michelle Wilson[1], Grant D. Stewart[2*], Adebanji Adeyoju[3], Jon Cartledge[4], Michael Kimuli[4], Shibendra Datta[5], Damian Hanbury[6], David Hrouda[7], Grenville Oades[8], Poulam Patel[9], Naeem Soomro[10], Mark Sullivan[11], Jeff Webster[12], Peter J. Selby[1], Rosamonde E. Banks[1]

[1]Leeds Institute of Medical Research at St James's, St. James's University Hospital, Beckett Street, Leeds LS9 7TF, UK.
[2]NHS Lothian, Edinburgh, UK.
[3]Stockport NHS Foundation Trust, Stockport, UK.
[4]Department of Urology, St James's University Hospital, Beckett Street, Leeds LS9 7TF, UK.
[5]University Hospital of Wales, Cardiff Heath Park, Cardiff, Wales CF14 4XW
[6]Lister Hospital, Stevenage SG1 4AB UK
[7]Charing Cross Hospital, Imperial College Healthcare NHS Trust, London W6 8RF
[8]Queen Elizabeth University Hospital, Glasgow G51 4TF UK
[9]Division of Cancer & Stem Cells, School of Medicine, University of Nottingham, Nottingham UK
[10]Newcastle Upon Tyne Hospitals NHS Foundation Trust, Newcastle upon Tyne, UK
[11]Oxford University Hospitals NHS Foundation Trust, Oxford UK
[12]Northwick Park Hospital Watford Road Harrow, HA1 3UJ UK
*Current affiliation: Department of Surgery, University of Cambridge, Cambridge Biomedical Campus, Addenbrooke's Hospital, Cambridge, CB2 0QQ, UK

Corresponding author: Dr N Vasudev, St. James's University Hospital, Beckett Street, Leeds LS9 7TF, UK. Tel: +44 113 2067680; Fax: +44 113 2429886; e-mail: n.vasudev@leeds.ac.uk

Key words: Diagnosis; Haematuria; Incidental; Presentation; Renal cell carcinoma; Symptoms

For peer review only

Abstract

Objectives: To describe the frequency and nature of symptoms in patients presenting with suspected renal cell carcinoma (RCC) and examine their reliability in achieving early diagnosis

Design: Multicentre prospective observational cohort study

Setting and Participants: Eleven UK centres recruiting patients presenting with suspected newly diagnosed RCC. Symptoms reported by patients were recorded and reviewed. Comprehensive clinico-pathological and outcome data were also collected.

Outcomes: Type and frequency of reported symptoms. Incidental diagnosis rate. Metastasis-free and cancer-specific survival.

Results: From 706 patients recruited between 2011-2014, 608 patients with a confirmed RCC formed the primary study population. The majority (60%) of patients were diagnosed incidentally. 87% of patients with stage Ia and 36% with stage III or IV disease presented incidentally. Visible haematuria was reported in 23% of patients and was commonly associated with advanced disease (49% had stage III or IV disease). Symptomatic presentation was associated with poorer outcomes, likely reflecting the presence of higher stage disease. Symptom patterns amongst the 54 patients subsequently found to have a benign renal mass were similar to those with a confirmed RCC.

Conclusions: Raising public awareness of RCC-related symptoms as a strategy to improve early detection rates is limited by the fact that related symptoms are relatively uncommon and often associated with advanced disease. Greater attention must be paid to the feasibility of screening strategies and the identification of circulating diagnostic markers.

For peer review only - http://bmjopen.bmj.com/site/about/guidelines.xhtml

Strengths and limitations of this study

- The multicentre, prospective nature of this study, amongst a contemporary cohort of UK patients, is unique and represents an important strength over previous studies
- Comprehensive linked clinico-pathological and outcome data was available for all patients
- Symptoms amongst patients subsequently found to have a benign renal mass are reported in parallel
- This was not a population-based study and our cohort represents only a small proportion of all patients diagnosed with RCC in the UK within the study period
- Patient reported symptoms were recorded following referral to secondary care and may therefore be subject to recall bias

For peer review only

Introduction

The incidence of kidney cancer in Europe is amongst the highest worldwide. In the UK, incidence rates have risen by 47% increase over the past decade, with 12,000 new cases in 2015 [1]. By 2035, it is predicted that this number will rise to over 20,000 new cases per annum and kidney cancer will come to represent the 4th commonest cancer amongst males and 9th commonest amongst females in the UK [2].

Diagnosing patients with kidney cancer can be challenging [3]. Renal cell carcinomas (RCCs), which make up the majority (85%) of kidney cancers, are characteristically insidious in onset. The once classical triad of haematuria, pain and abdominal mass is now recognised to be rare and symptoms, if present at all, can be vague, non-specific and delayed in onset. Whilst early diagnosis is recognised to be key in achieving optimal outcomes, many patients still present with advanced disease. In 2017 in England, for example, figures show that amongst patients with a recorded stage at diagnosis, 19% had stage III and 23% had stage IV disease, at the time of presentation [4].

Campaigns to raise awareness of kidney cancer amongst the public and doctors have been employed in an effort to improve early diagnosis rates [5]. Understanding how patients present may help to inform such strategies. Unlike previous studies, we prospectively collected information on symptoms reported by patients at the time of their diagnosis of suspected RCC, following recruitment to a large, contemporary, multi-institutional UK RCC biobank [6]. The aims of this sub-study were to describe symptoms reported by patients, define the current rate of incidental diagnosis and

For peer review only

look at how these factors relate to patient outcomes, with the goal of better understanding the challenges in early RCC diagnosis.

Methods

The design was a multicentre prospective observational cohort study. Patients with a renal mass on imaging suspicious of RCC, of all stages, with no prior treatment, were eligible. Patients were approached and consented to participate in the study prior to surgery or biopsy, before diagnosis of RCC was confirmed. Full details regarding the inclusion and exclusion criteria are as previously reported [6]. Comprehensive clinical and pathological information was collected.

At the time of recruitment to the study, patients were asked about the presence and nature of symptoms leading to their diagnosis of suspected RCC, which was recorded using paper case-report forms (CRF). Specific questions relating to commonly related 'RCC-type' local symptoms (pain, haematuria, abdominal mass and/or other) and/or systemic symptoms (weight loss (any), loss of appetite, sweats, fevers, fatigue and/or other) were recorded. In addition, the investigator completing the CRF was asked to state whether the diagnosis was incidental in nature and included a subsequent free-text box requesting a description of how the patient was diagnosed. All cases were independently reviewed by two reviewers (NV and RB) to confirm or refute whether the diagnosis would be regarded as incidental or not (i.e. were any symptoms reported and, if so, would they be regarded as being related to the finding of RCC), with additional reference to individual electronic case notes where available. Reported presence of RCC-type symptoms, many of which, such as pain, are non-specific, was not always related to the finding of RCC and, where

For peer review only - http://bmjopen.bmj.com/site/about/guidelines.xhtml

applicable therefore, considered incidental. Cases with insufficient data or where the incidental nature of the diagnosis remained uncertain were not classified. Patients being investigated for asymptomatic hypertension were not classified as incidental [7].

Metastasis-free survival (MFS) was calculated for patients with localised disease, defined as the period from date of nephrectomy to date of distant recurrence. Patients without recurrence were censored at the date they were last known to be recurrence-free (for patients who died without recurrence this was date of death). Cancer-specific survival (CSS) was defined as the period from date of nephrectomy to the date of cancer-related death. Patients with a non-cancer related death were censored at their date of death and patients still alive were censored at the last date they were known to be alive. Kaplan-Meier plots were produced to visualise survival and the log-rank test was used to detect statistically significant difference between survival curves.

Public and Patient Involvement

Patients were extensively involved in the design, delivery and evaluation of the NIHR Programme supporting this work. Patients were not directly involved in the design or evaluation of the current report.

For peer review only

For peer review only - http://bmjopen.bmj.com/site/about/guidelines.xhtml

Results

Between July 2011 and June 2014, 706 patients were recruited to the study from 11 UK centres (8 England; 2 Scotland; 1 Wales). Details regarding recruitment by centre are shown in **Supplementary Table 1**. The flow of patients through the study is shown in **Figure 1**. RCC was confirmed in 608 (86%) patients, amongst whom median follow-up was 4.8 yrs (IQR: 3.7, 5.2), and benign renal mass in 54 (7.6%) patients. The remaining 44 (6.4%) patients either did not undergo biopsy or nephrectomy or had no tumour in their biopsy cores (n=33), had another (not RCC) malignancy (n=5), or an alternative benign pathology (n=6) [6]. Amongst all patients with a confirmed RCC, 422 (69%) patients reported having RCC-type symptoms at diagnosis, of whom 221 (52%) reported symptoms that were considered related to the presence of RCC. Amongst these 221 patients, 97 (44%) had local symptoms only, 19 (8.6%) had systemic symptoms only and 105 (47.5%) reported having both local and systemic symptoms. Patient and tumour characteristics by symptom type are shown in **Table 1**.

Local RCC-related symptoms

Amongst the 202 (33%) patients reporting local RCC-related symptoms, 137 (68%) reported visible haematuria and 126 (62%) reported pain, with only 14 (7%) patients reporting an abdominal mass. Patients presenting with haematuria had a median pathological tumour size of 75mm (range 16-155) and almost half had stage III (37.2%) or IV (12.4%) disease. Only four patients (0.6%) presented with the classical triad of an abdominal mass, haematuria and local pain. The median tumour size amongst these four patients was 105 mm (range 80-154 mm) on preoperative cross-sectional imaging. No significant differences were present when considered by

For peer review only - http://bmjopen.bmj.com/site/about/guidelines.xhtml

histological type, although the small number of patients with non-clear cell RCC limits this comparison.

Systemic RCC-related symptoms

Amongst those reporting systemic symptoms related to their RCC, fatigue (62%), weight loss (52%), sweats (38%) and loss of appetite (38%) were all commonly reported. Fever was relatively uncommon (10%). Patients with systemic symptoms were more likely to have grade 4 cancers and stage IV disease than those with local RCC-related symptoms only and those with symptoms unrelated to RCC ($p<0.01$) (**Table 1**).

Incidental diagnosis

Amongst the 582 patients in whom the nature of the diagnosis could be confidently classified, 351 (60%) cases of RCC were deemed to have been diagnosed incidentally. Patient and tumour characteristics by nature of diagnosis (incidental vs non-incidental) are shown in **Table 2**. No association with patient sex was found and distribution of histological subtype was similar between groups. Non-incidentally detected tumours were larger and of higher grade and stage than incidentally detected tumours ($p<0.01$). Amongst patients diagnosed with a localised pT1a tumour, the incidental diagnosis rate was 87%. Conversely, 22% of patients with stage IV disease were considered to have been diagnosed incidentally. The nature of the incidental diagnosis (e.g. during investigation for a known pre-existing condition versus investigation of unrelated symptoms) is shown in **Table 3**.

Tumour size

For peer review only - http://bmjopen.bmj.com/site/about/guidelines.xhtml

Pathological tumour size was available for 556 (91%) of patients. We looked at symptoms in patients presenting with tumours ≥10 cm. Amongst the 66 patients with a tumour ≥10cm, 31 (47%) reported haematuria at the time of presentation, 33 (50%) reported pain, and abdominal mass was reported in four (6%) patients. Almost a quarter (16/66; 24%) of these patients were considered to have been diagnosed incidentally, with 10 (15%) reporting no symptoms, despite the presence of a large primary tumour. No effect of BMI was observed in relation to presence or absence of symptoms.

Outcomes

We looked at survival outcomes by both symptom type (no RCC-type symptoms or unrelated RCC-type symptoms vs. related RCC-type symptoms) and incidental versus non-incidental diagnosis. Patients diagnosed with no RCC-type symptoms and those reporting unrelated RCC-type symptoms had a significantly improved MFS and CSS compared to patients with related RCC-type symptoms. Furthermore, patients with systemic RCC-related symptoms had poorer outcomes than those with local RCC symptoms only (**Figure 2 A and B**). Overall, patients with an incidental diagnosis of RCC had improved MFS and CSS in comparison to those diagnosed non-incidentally, although it is important to note that these effects were lost when controlled for stage of disease (**Figure 3**).

Patients presenting with benign renal masses

In total, 54 (7.6%) patients in our cohort were found to have a benign renal mass, composed of oncocytoma (n=29), angiomyolipoma (n=8) and other lesions (n=17) (**Table 4**). The incidental diagnosis rate was 56% amongst the 52 evaluable patients.

For peer review only - http://bmjopen.bmj.com/site/about/guidelines.xhtml

Haematuria and pain were reported in 57% and 52% of patients diagnosed non-incidentally. The majority (65%) reported symptoms, of whom 57% had local symptoms only, 17% had systemic symptoms only and 26% reported both local and systemic symptoms.

Discussion

Early detection is widely held to be a key strategy towards improving outcomes in patients with RCC [8]. As in most solid cancers, disease stage and survival are closely linked, with 3-year CSS rates in our cohort for example, of 99% and 47% for stage I and stage IV cancers, respectively (data not shown). Symptoms of kidney cancer such as visible haematuria and flank pain are well documented and NHS initiatives such as 'be clear on cancer: blood in your pee' campaign have been aimed at prompting the public to seek early medical attention [5]. Nevertheless, many patients still present with overt or micro-metastatic disease. Understanding the type and frequency of symptoms patients with newly diagnosed RCC report is critical in beginning to address this issue and understand whether simply raising awareness amongst doctors and the public is sufficient or other strategies are needed.

Our study highlights the significant challenges in diagnosing patients with kidney cancer. Almost a third of patients in our cohort were symptomless at the time of diagnosis, amongst whom nearly a quarter (24%) had stage III or IV disease. Visible haematuria, a hallmark symptom of this disease, was recorded in just 23% of patients overall. Even amongst patients with large (≥10cm) tumours, less than half (47%) reported haematuria as a symptom. Prior reports using UK general practice database records have suggested rates of haematuria as low as 18% in patients

For peer review only - http://bmjopen.bmj.com/site/about/guidelines.xhtml

presenting with kidney cancer, compounded by the low positive predictive value (PPV) (1%) of this symptom for RCC amongst those ≥60 yr old [9]. Furthermore, symptom patterns do not appear to reliably distinguish patients with benign renal masses from those with RCC.

Many studies have attempted to document the incidental diagnosis rate for renal cancer. These previous studies have all been retrospective in nature, typically derived from patients at a single centre, with widely varying rates of incidental diagnosis, from 15% to 61%, in a less contemporaneous setting (broadly spanning 1970-2000) [10-14]. A more recent, global, study, involving 4288 patients presenting with RCC between 2010-2012, reported an incidental diagnosis rate of 67% [15]. However, no detail regarding how this was derived, or the nature and characteristics of those diagnosed incidentally were presented in this study. Whilst retrospective studies have the advantage of being feasible on a large scale, often with long term follow-up data, recording of symptoms at presentation may not have been performed for this purpose and may, therefore, not be complete. Furthermore, determining whether a diagnosis is incidental or not can often require further detail beyond the recording of symptoms alone, and which may not always be available when records are reviewed retrospectively. Here, we collected symptoms reported by patients at diagnosis in a planned way as part of the study design using standardised CRFs, allowed for detailed free-text annotation of the history leading to the diagnosis and asked investigators to specifically indicate whether this was felt to be incidental in nature. We carefully reviewed the presenting symptoms and history for each patient in our study, performed independently by two of the authors, to determine as accurately as possible whether the diagnosis would be deemed incidental or not.

For peer review only - http://bmjopen.bmj.com/site/about/guidelines.xhtml
For peer review only

Pain, for example, was a commonly reported symptom not necessarily attributable to the diagnosis of RCC, for example when located in an anatomically distinct site. We believe our figure of 60%, amongst a contemporary set of patients (2011-2014), provides a true reflection of the current incidental diagnosis rate of RCC in the UK, and supports the general rise in the incidental detection of kidney cancer that has been reported over time.

Our data shows that the majority (60%) of patients with RCC in the UK are being diagnosed incidentally, with almost three-quarters of these (74%) during investigation of symptoms unrelated to RCC. By contrast, a Norwegian study of 413 patients diagnosed with RCC between 1997-2010 reported a 53% incidental diagnosis rate, detected in 63% of these patients during follow-up for a pre-existing condition [16]. The reason for this difference is not certain but may reflect the different time periods under study, given the more liberal use of cross-sectional imaging over time [17]. Consistent with other studies, patients with an incidentally detected RCC tended to have smaller, lower stage and grade tumours than those presenting with related symptoms, but, nevertheless, almost one in five of patients identified incidentally had stage III/IV disease at diagnosis. Whether patients who are diagnosed incidentally have better outcomes and potentially, therefore, different tumour biology, than those presenting with symptoms has been a matter of debate in the literature [10,18-20]. We did not find any difference in MFS or CSS between these two groups when matched for stage of disease, suggesting that incidental detection of advanced stage disease is not advantageous in terms of outcome.

For peer review only - http://bmjopen.bmj.com/site/about/guidelines.xhtml

Diagnosing kidney cancer early is therefore a significant public health challenge. Data from the 2010 National Cancer Patient Experience Survey in England report that almost 30% of 564 patients with renal cancer saw their general practitioner three or more times before hospital referral [21]. Furthermore, results from the charity Kidney Cancer UK (KCUK) 2018 patient survey showed that 22% of the 153 responders who presented to their GP or an A+E department waited more than 3 months for a diagnosis [22]. The results of the KCUK survey (n=175 in total) extend further, with 51% of patients reporting their cancer being detected incidentally during imaging for an unrelated reason, and less than one third (31%) having symptoms due to RCC, reflecting the findings from our own, much larger, study.

How then do we improve rates of early diagnosis in kidney cancer? Raising awareness amongst the public to present early to their doctor, even with vague symptoms, may seem logical, as well as increasing awareness with primary care teams. But many patients remain asymptomatic until they have advanced stage disease, and the PPVs for symptoms other than haematuria, such as pain and fatigue, are even lower than 1% [9], placing an impossible demand on general practitioners, who are required to act as gatekeepers to secondary care. Five-year survival rates for kidney cancer in the UK lag behind the European average which may be related to differences in stage at diagnosis [23]. Greater availability of point-of-care ultrasound may make a significant impact but its use varies widely across Europe and has not been widely adopted in the UK, with potential barriers in terms of time and training [24].

For peer review only

For peer review only - http://bmjopen.bmj.com/site/about/guidelines.xhtml

Interest in exploring the potential for kidney cancer screening is growing [8,25], particularly given the significant predicted rise in incidence [2]. The potential cost-effectiveness of performing a single, renal focused, USS amongst asymptomatic 60-year-old men has recently been reported [26]. However numerous uncertainties still exist, in terms of who to screen, with what modality, as well as unknowns in terms of associated harms versus benefit [27]. This is an area that clearly warrants further research. The identification of robust diagnostic biomarkers either in the serum or urine of patients that could be used to easily rule in or out the presence of RCC is another priority area for study [28], with recent promising reports in the literature [29], although still requiring significant further validation and improved performance.

The strengths of this study include its prospective multicentre design, amongst a contemporary cohort of patients with robust linked clinicopathological and outcome data. The eligibility criteria for the study were broad and we believe our patient cohort to be largely representative, when considered at a population level (for comparisons by age, sex, stage and RCC type see **Supplementary Table 2**). It is possible that the proportion of patients in our study with stage IV disease may be slightly lower than in the true population, reflecting differences in the clinical pathway these patients may take, which may have impacted on our reported rate of incidental diagnosis. Furthermore, not all patients seen at participating centres with suspected RCC during the study period were recruited to the study and, overall, we acknowledge that our cohort size reflects only a small proportion (less than 10%) of all patients diagnosed with RCC in the UK during the study period. A further limitation is the fact that patient-reported symptoms were recorded following referral to secondary care and there may, therefore, be some element of recall bias.

For peer review only - http://bmjopen.bmj.com/site/about/guidelines.xhtml

In summary, this study draws attention to the fact that reliance on symptoms for the early detection of kidney cancer is not robust. Our data suggest that improving public and professional awareness will have only a limited impact, and innovative biomarkers for this purpose remain to be identified. We suggest it is time to re-examine the case for screening looking at opportunities to link RCC screening into other programmes such as low dose CT scans for lung cancer health checks or ultrasound-based screening for abdominal aortic aneurysms.

Contributors: Funding: PJS and REB; Study design and concept: PJS, REB, NSV; Patient recruitment and data collection: AA, JC, MK, SD, DHa, DHr, GO, PP, NS, GDS, MS, JW, NSV, REB; Data analysis: MW, GDS, NSV, REB; Manuscript preparation: NSV, MW, GDS, PJS, REB. All authors participated in the interpretation of results, critically revised the paper and approved the final version to be published.

Ethics approval and consent to participate: The study was approved by the Leeds East Research Ethics Committee (ethical approval 10/H1306/6). Patients were recruited to the study with their written informed consent

Funding: This project was funded by the National Institute for Health Research Programme Grants for Applied Research programme (project number NIHR PGfAR RP-PG-0707-10101) and has been published in full in Programme Grants for Applied Research Volume: 6, Issue: 3. Further information is available at: https://www.journalslibrary.nihr.ac.uk/pgfar/pgfar06030/#/abstract. Additional support was provided by the NIHR Leeds Clinical Research Facility. The views and opinions expressed by the authors in this publication are those of the authors and do not necessarily reflect those of the NHS, the NIHR or the Department of Health.

Disclaimer: The funder had no role in study design, data collection, data analysis or manuscript preparation

Conflicts of interest: N.S.V. discloses paid consultancy work and/or speaker honoraria for EUSA pharma, Bristol Myers Squibb, Pfizer, Merck and Novartis. G.D.S. discloses paid consultancy work for Pfizer, EUSA Pharma, Merck, and CMR Surgical and an advisory board role for the latter. The other authors declare no conflicts of interest.

Acknowledgements: We are grateful to the patients, the staff at participating centres who participated in patient recruitment and data collection, staff within the Leeds Biobanking and Sample Processing Lab and staff at the Leeds Clinical Trials Research Unit.

For peer review only - http://bmjopen.bmj.com/site/about/guidelines.xhtml

Patient consent for publication: Not required

Data availability statement: All data relevant to the study are included in the article

References

1. Cancer Research UK. https://www.cancerresearchuk.org/health-professional/cancer-statistics-for-the-uk. Accessed August 2019.
2. Smittenaar CR, Petersen KA, Stewart K, Moitt N. Cancer incidence and mortality projections in the UK until 2035. *Br J Cancer* 2016:**115**:1147-55
3. Zhou Y, van Melle M, Singh H, Hamilton W, Lyratzopoulos G, Walter FM. Quality of the diagnostic process in patients presenting with symptoms suggestive of bladder or kidney cancer: a systematic review. *BMJ Open* 2019:**9**:e029143
4. http://ncin.org.uk/publications/survival_by_stage. Accessed August 2019
5. https://www.nhs.uk/be-clear-on-cancer/symptoms/blood-in-pee. Accessed August 2019
6. Vasudev NS, Hutchinson M, Trainor S *et al.* UK multicentre prospective evaluation of the Leibovich score in localised renal cell carcinoma: performance has altered over time. *Urology* 2019. In Press
7. Chow WH, Gridley G, Fraumeni JF, Jr., Jarvholm B. Obesity, hypertension, and the risk of kidney cancer in men. *N Engl J Med* 2000:**343**:1305-11
8. Motzer RJ. Perspective: What next for treatment? *Nature* 2016:**537**:S111
9. Shephard E, Neal R, Rose P, Walter F, Hamilton WT. Clinical features of kidney cancer in primary care: a case-control study using primary care records. *Br J Gen Pract* 2013:**63**:e250-5
10. Tsui KH, Shvarts O, Smith RB, Figlin R, de Kernion JB, Belldegrun A. Renal cell carcinoma: prognostic significance of incidentally detected tumors. *J Urol* 2000:**163**:426-30
11. Lee CT, Katz J, Fearn PA, Russo P. Mode of presentation of renal cell carcinoma provides prognostic information. *Urol Oncol* 2002:**7**:135-40
12. Ficarra V, Prayer-Galetti T, Novella G *et al.* Incidental detection beyond pathological factors as prognostic predictor of renal cell carcinoma. *Eur Urol* 2003:**43**:663-9
13. Luciani LG, Cestari R, Tallarigo C. Incidental renal cell carcinoma-age and stage characterization and clinical implications: study of 1092 patients (1982-1997). *Urology* 2000:**56**:58-62
14. Jayson M, Sanders H. Increased incidence of serendipitously discovered renal cell carcinoma. *Urology* 1998:**51**:203-5
15. Laguna MP, Algaba F, Cadeddu J *et al.* Current patterns of presentation and treatment of renal masses: a clinical research office of the endourological society prospective study. *J Endourol* 2014:**28**:861-70

For peer review only - http://bmjopen.bmj.com/site/about/guidelines.xhtml

16. Sand KE, Hjelle KM, Rogde AJ, Gudbrandsdottir G, Bostad L, Beisland C. Incidentally detected renal cell carcinomas are highly associated with comorbidity and mortality unrelated to renal cell carcinoma. *Scand J Urol* 2013:**47**:462-71
17. https://www.gov.uk/government/publications/review-of-radiation-dose-issues-from-the-use-of-ct-in-the-uk. Accessed August 2019
18. Patard JJ, Rodriguez A, Rioux-Leclercq N, Guille F, Lobel B. Prognostic significance of the mode of detection in renal tumours. *BJU Int* 2002:**90**:358-63
19. Palsdottir HB, Hardarson S, Petursdottir V *et al.* Incidental detection of renal cell carcinoma is an independent prognostic marker: results of a long-term, whole population study. *J Urol* 2012:**187**:48-53
20. Gudbjartsson T, Thoroddsen A, Petursdottir V, Hardarson S, Magnusson J, Einarsson GV. Effect of incidental detection for survival of patients with renal cell carcinoma: results of population-based study of 701 patients. *Urology* 2005:**66**:1186-91
21. Lyratzopoulos G, Neal RD, Barbiere JM, Rubin GP, Abel GA. Variation in number of general practitioner consultations before hospital referral for cancer: findings from the 2010 National Cancer Patient Experience Survey in England. *Lancet Oncol* 2012:**13**:353-65
22. https://www.kcuk.org.uk/patient-survey-2018/. Accessed August 2019
23. De Angelis R, Sant M, Coleman MP *et al.* Cancer survival in Europe 1999-2007 by country and age: results of EUROCARE--5-a population-based study. *Lancet Oncol* 2014:**15**:23-34
24. Mengel-Jorgensen T, Jensen MB. Variation in the use of point-of-care ultrasound in general practice in various European countries. Results of a survey among experts. *Eur J Gen Pract* 2016:**22**:274-7
25. Shea MW. A proposal for a targeted screening program for renal cancer. *Front Oncol* 2013:**3**:207
26. Rossi SH, Klatte T, Usher-Smith JA *et al.* A Decision Analysis Evaluating Screening for Kidney Cancer Using Focused Renal Ultrasound. *Eur Urol Focus* 2019
27. Rossi SH, Klatte T, Usher-Smith J, Stewart GD. Epidemiology and screening for renal cancer. *World J Urol* 2018:**36**:1341-53
28. Capitanio U, Bensalah K, Bex A *et al.* Epidemiology of Renal Cell Carcinoma. *Eur Urol* 2019:**75**:74-84
29. Morrissey JJ, Mellnick VM, Luo J *et al.* Evaluation of Urine Aquaporin-1 and Perilipin-2 Concentrations as Biomarkers to Screen for Renal Cell Carcinoma: A Prospective Cohort Study. *JAMA Oncol* 2015:**1**:204-12

For peer review only - http://bmjopen.bmj.com/site/about/guidelines.xhtml

Figure Legends

Figure 1. Flow of patients through the study

Figure 2. Kaplan Meier survival curves by symptom type. Survival outcomes (A. MFS; B. CSS) in patients with no RCC-type symptoms, unrelated RCC-type symptoms, local RCC-related symptoms and those with systemic (+/- local) RCC-related symptoms

Figure 3. Kaplan Meier survival curves by incidental vs. non-incidental diagnosis for all patients, stage I/II or stage III RCC. A-C: MFS; D-F: CSS

For peer review only

For peer review only - http://bmjopen.bmj.com/site/about/guidelines.xhtml

Table 1. Patient and tumour characteristics by symptom type
For continuous variables, figures in table represent median (range) with corresponding p-value from the Kruskal-Wallis test and for categorical variables, figures in table represent n (%) with corresponding p-value from chi-squared test.

		RCC-type symptoms reported (n=422)**			
Characteristic	No RCC-type symptoms (n=186)	Not RCC related (n=183)	RCC-related local symptoms only (n=97)	RCC-related systemic symptoms (+/- local) (n=124)	p-value
**Age (years)**	65 (31-86)	63 (29-90)	63 (38-84)	62 (33-92)	0.31
**Gender**					
**Female**	67 (32.7)	62 (30.2)	21 (10.2)	55 (26.8)	
**Male**	119 (30.9)	121 (31.4)	76 (19.7)	69 (17.9)	0.01
**BMI**	28.5 (15.6-74.4)	27 (18.1-56.5)	28.8 (17.3-67.2)	27.5 (16-54.5)	0.01
**Tumour size (mm)**	44 (14-180)	43 (11-170)	74 (13-155)	75 (20-240)	<0.01
**pT**					
**1a**	83 (42.6)	88 (45.1)	16 (8.2)	8 (4.1)	
**1b**	46 (34.3)	42 (31.3)	19 (14.2)	27 (20.1)	
**2**	15 (19.7)	18 (23.7)	19 (25)	24 (31.6)	
**3**	38 (22.6)	33 (19.6)	42 (25)	55 (32.7)	
**4**	0 (0)	0 (0)	1 (25)	3 (75)	
**X**	1 (50)	1 (50)	0 (0)	0 (0)	
**Missing**	1 (100)	0 (0)	0 (0)	0 (0)	
**NA**	2 (20)	1 (10)	0 (0)	7 (70)	<0.01
**Grade**					
**1**	4 (40)	0 (0)	4 (40)	2 (20)	
**2**	55 (34.8)	50 (31.6)	25 (15.8)	28 (17.7)	
**3**	88 (32.2)	94 (34.4)	47 (17.2)	44 (16.1)	
**4**	13 (14.9)	13 (14.9)	19 (21.8)	42 (48.3)	
**Missing**	9 (39.1)	9 (39.1)	1 (4.3)	4 (17.4)	
**NA**	17 (43.6)	17 (43.6)	1 (2.6)	4 (10.3)	<0.01
**Stage**					
**I**	130 (39.8)	129 (39.4)	34 (10.4)	34 (10.4)	
**II**	12 (17.4)	17 (24.6)	18 (26.1)	22 (31.9)	
**III**	34 (24.5)	29 (20.9)	37 (26.6)	39 (28.1)	
**IV**	10 (18.9)	6 (11.3)	8 (15.1)	29 (54.7)	
**Missing**	0 (0)	2 (100)	0 (0)	0 (0)	<0.01
**Tumour subtype**					
**Clear Cell**	147 (31.7)	137 (29.6)	83 (17.9)	96 (20.7)	
**Papillary**	16 (27.1)	23 (39)	7 (11.9)	13 (22)	
**Chromophobe**	15 (32.6)	15 (30.4)	7 (15.2)	10 (21.7)	
**Unclassified**	7 (38.9)	6 (33.3)	0 (0)	5 (27.8)	
**Other**	1 (33)	2 (67)	0 (0)	0 (0)	0.81

*NA=not applicable - patients underwent biopsy only or tumour ablation
**18 patients reported symptoms but their relationship to RCC could not be determined

For peer review only - http://bmjopen.bmj.com/site/about/guidelines.xhtml

Table 2. Patient and tumour characteristics by diagnosis type
For continuous variables, figures in table represent median (range) with corresponding p-value from the Wilcoxon rank-sum test and for categorical variables, figures in table represent n (%) with corresponding p-value from chi-squared test.

Characteristic	Non-incidental (n=231)	Incidental (n=351)	p-value
**Age (years)**	62 (33-92)	65 (29-90)	0.04
**Gender**			
**Female**	77 (38.3)	124 (61.7)	
**Male**	154 (40.4)	227 (59.6)	0.69
**BMI**	28.3 (15.6-67.2)	27.8 (17.2-57.7)	0.38
**Tumour size (path) (mm)**	75 (13-240)	42 (11-170)	<0.01
**Tumour size (CT) (mm)**	80 (16-250)	44 (10-170)	<0.01
**pT**			
**1a**	25 (12.8)	170 (87.2)	
**1b**	48 (37.2)	81 (62.8)	
**2**	46 (60.5)	30 (39.5)	
**3**	101 (61.2)	64 (38.8)	
**4**	4 (100)	0 (0)	
**X**	0 (0)	2 (100)	
**Missing**	0 (0)	1 (100)	
**NA***	7 (70)	3 (30)	<0.01
**Grade**			
**1**	6 (66.7)	3 (33.3)	
**2**	56 (35.9)	100 (64.1)	
**3**	93 (34.6)	176 (65.4)	
**4**	65 (75.6)	21 (24.4)	
**Missing**	6 (26.1)	17 (73.9)	
**NA***	5 (12.8)	34 (87.2)	<0.01
**Stage**			
**I**	70 (21.6)	254 (78.4)	
**II**	42 (60.9)	27 (39.1)	
**III**	80 (58.4)	57 (41.6)	
**IV**	39 (78)	11 (22)	
**Missing**	0 (0)	2 (100)	<0.01
**Tumour subtype**			
**Clear Cell**	186 (40.9)	269 (59.1)	
**Papillary**	21 (35.6)	38 (64.4)	
**Chromophobe**	19 (41.3)	28 (58.7)	
**Unclassified**	5 (27.8)	13 (72.2)	
**Other**	0 (0)	3 (100)	0.62

*NA=not applicable, patients underwent biopsy only or tumour ablation

For peer review only - http://bmjopen.bmj.com/site/about/guidelines.xhtml

Table 3. Nature of incidental diagnosis

Type of incidental diagnosis	n (%)
**Investigation for pre-existing condition**	65 (18)
Another malignancy	34 (53)
Diabetes Mellitus	7 (11)
Hepatobiliary[a]	5 (8)
AAA screening / Post-aortic repair	3 (5)
Other[b]	16 (23)
**Investigation for signs or symptoms unrelated to RCC**	258 (74)
Gastrointestinal[c]	86 (33)
Urinary tract[d]	49 (19)
Hepatobiliary[e]	27 (10)
Respiratory[f]	20 (8)
Musculoskeletal[g]	16 (6)
Cardiovascular[h]	11 (4)
Trauma	7 (3)
Gynaecological	6 (3)
Anaemia	4 (2)
Miscellaneous[i]	32 (12)
**Routine health check**[k]	16 (5)
**Not known**[l]	12 (3)

AAA, abdominal aortic aneurysm; [a]cirrhosis, primary biliary cirrhosis, sclerosing cholangitis; [b]includes Addison's disease, chronic renal failure, crohn's disease, coeliac disease, ovarian cyst, renal stones, IgA nephropathy, Wegener's granulomatosis, polymyalgia rheumatica, ovarian cyst; [c]altered bowel habit, GI bleed, bloating/distension, abdominal pain, reflux; [d]urinary retention, prostatic symptoms, high PSA, urosepsis, renal colic, impaired renal function; [e]biliary colic, deranged liver function tests, jaundice, pancreatitis, cholecystitis; [f]shortness of breath, cough, haemoptysis, pneumonia; [g]back pain, leg pain, joint pain; [h]chest pain, myocardial infarction, claudication, endocarditis; [i]includes dizziness, syncope, elevated blood test values, ankle swelling; [k]Initial investigations were urine dip (6), USS (5), CT scan (2), blood tests (2), CXR (1); [l]insufficient information to classify

For peer review only - http://bmjopen.bmj.com/site/about/guidelines.xhtml

Table 4. Characteristics and symptoms associated with benign renal masses

Characteristic	All (n=54)	Oncocytoma (n=29)	AML (n=8)	Other* (n=17)
**Age (years)**	65 (32-86)	66 (42-86)	63 (59-68)	61 (32-78)
**Gender**				
**Female**	29 (53.7)	12 (41.4)	5 (62.5)	12 (70.6)
**Male**	25 (46.3)	17 (58.6)	3 (37.5)	5 (29.4)
**BMI**	27.6 (18.7-45.8)	27.8 (19.4-39.6)	28 (22-38.8)	26.4 (18.7-45.8)
**CT size (cm)**				
**≤4**	22 (44.9)	14 (50)	3 (50)	5 (33.3)
**4< - ≤7**	18 (36.7)	11 (39.3)	2 (33.3)	5 (33.3)
**7< - ≤10**	6 (12.2)	1 (3.6)	1 (16.7)	4 (26.7)
**>10**	3 (6.1)	2 (7.1)	0 (0)	1 (6.7)
**NA**	5 (-)	1 (-)	2 (-)	2 (-)
**RCC-type symptoms**				
**No**	19 (35.2)	10 (34.5)	4 (50)	5 (29.4)
**Yes**	35 (64.8)	19 (65.5)	4 (50)	12 (70.6)
**Local symptoms**				
**No**	6 (17.1)	3 (15.8)	1 (25)	2 (16.7)
**Yes**	29 (82.9)	16 (84.2)	3 (75)	10 (83.3)
**Systemic symptoms**				
**No**	20 (57.1)	12 (63.2)	1 (25)	7 (58.3)
**Yes**	15 (42.9)	7 (36.8)	3 (75)	5 (41.7)
**Incidental diagnosis**				
**No**	23 (42.5)	13 (44.8)	2 (25)	8 (47)
**Yes**	29 (54)	15 (51.7)	6 (75)	8 (47)
**Not known**	2 (3.5)	1 (3.5)	0 (0)	1 (6)

AML – angiomyolipoma *consists of cystic nephroma (4), benign cyst (3), metanephric adenoma (2), mixed epithelial stromal tumour (2), haemangioblastoma (1), leiomyomata (1), multilocular cyst (1), myxoid mesenchymal tumour (1), Rosai Dorfman disease (1), solitary fibrous tumour (1)

For peer review only

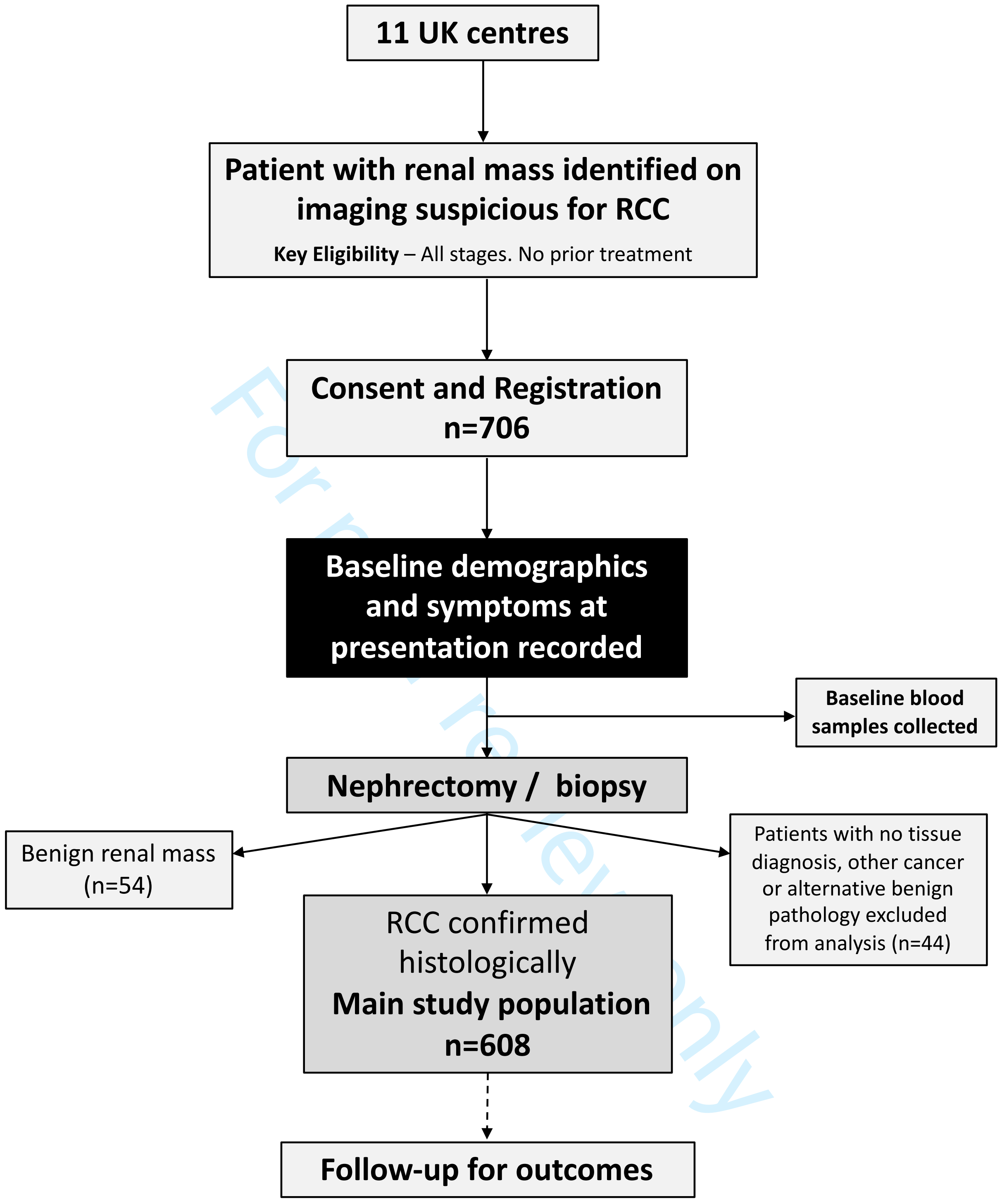

For peer review only

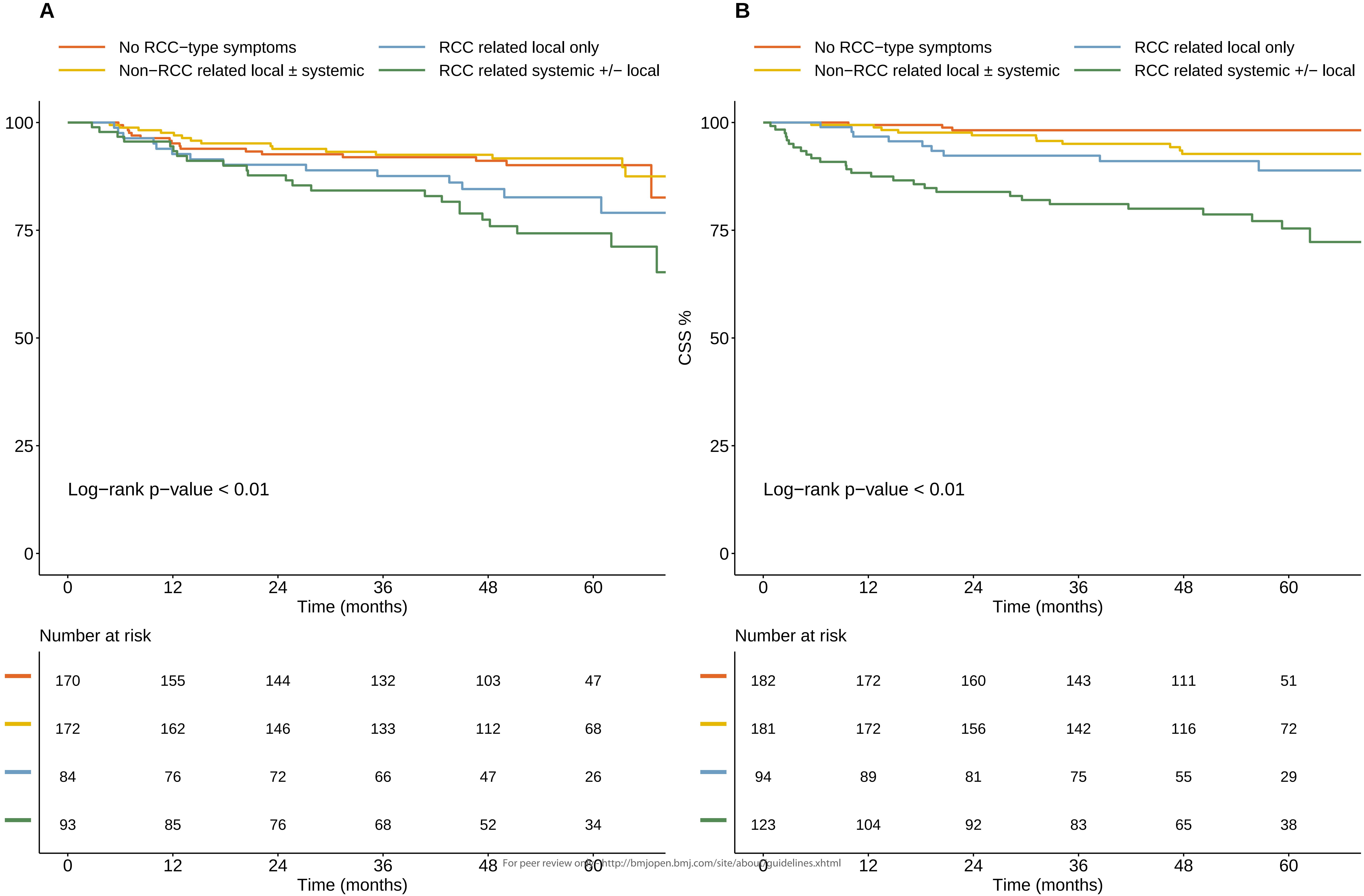
A
No RCC−type symptoms
Non−RCC related local ± systemic
RCC related local only
RCC related systemic +/− local
MFS %
100
75
50
25
0
Log−rank p−value < 0.01
0 12 24 36 48 60
Time (months)
Number at risk
170 155 144 132 103 47
172 162 146 133 112 68
84 76 72 66 47 26
93 85 76 68 52 34
0 12 24 36 48 60
Time (months)
B
No RCC−type symptoms
Non−RCC related local ± systemic
RCC related local only
RCC related systemic +/− local
CSS %
100
75
50
25
0
Log−rank p−value < 0.01
0 12 24 36 48 60
Time (months)
Number at risk
182 172 160 143 111 51
181 172 156 142 116 72
94 89 81 75 55 29
123 104 92 83 65 38
0 12 24 36 48 60
Time (months)

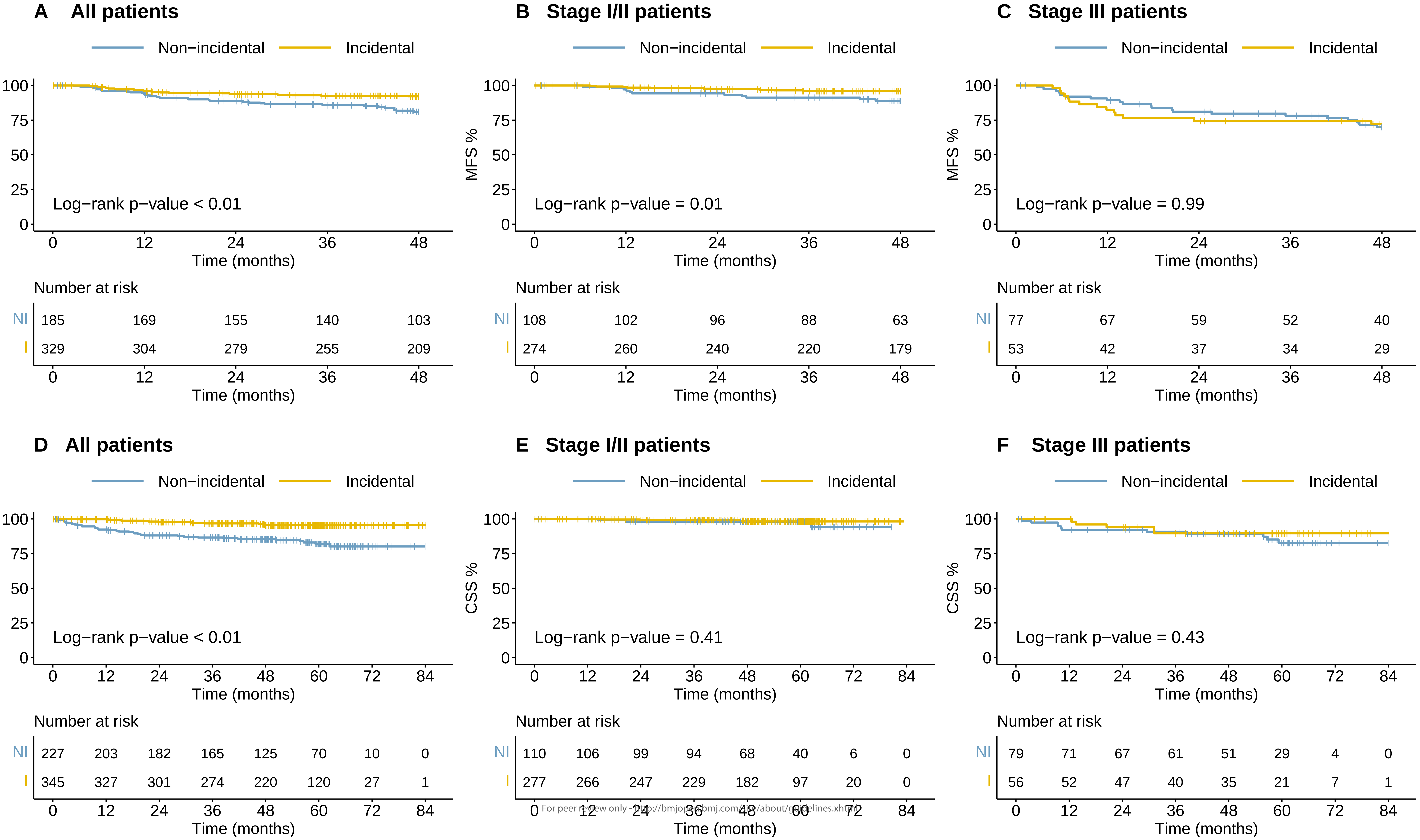
A All patients
Non−incidental
Incidental
MFS %
Log−rank p−value < 0.01
Time (months)
Number at risk
NI 185 169 155 140 103
I 329 304 279 255 209
B Stage I/II patients
MFS %
Log−rank p−value = 0.01
Number at risk
NI 108 102 96 88 63
I 274 260 240 220 179
C Stage III patients
MFS %
Log−rank p−value = 0.99
Number at risk
NI 77 67 59 52 40
I 53 42 37 34 29
D All patients
CSS %
Log−rank p−value < 0.01
Number at risk
NI 227 203 182 165 125 70 10 0
I 345 327 301 274 220 120 27 1
E Stage I/II patients
CSS %
Log−rank p−value = 0.41
Number at risk
NI 110 106 99 94 68 40 6 0
I 277 266 247 229 182 97 20 0
F Stage III patients
CSS %
Log−rank p−value = 0.43
Number at risk
NI 79 71 67 61 51 29 4 0
I 56 52 47 40 35 21 7 1

Supplementary Table 1. Study recruitment by centre

Centre ID	Total patients recruited n (% of total)	Recruitment period (months)*	Recruitment rate (pt/mo)
50	255 (36.1)	35	7.3
69	103 (14.6)	19	5.4
221	75 (10.6)	28	2.7
361	72 (10.3)	23	3.1
15	62 (8.8)	21	2.9
39	44 (6.2)	25	1.8
153	33 (4.7)	15	2.2
352	27 (3.8)	25	1.1
537	13 (1.8)	12	1.1
132	13 (1.8)	15	0.9
131	9 (1.3)	11	0.8

First patient registered July 2011; Last patient registered June 2014
* recruitment times vary based on the fact that centres opened to recruitment at different times

For peer review only

Supplementary Table 2. Comparison of current study patient characteristics with other RCC populations

Characteristic	Current Cohort (n=608)	UK RCC population data	US RCC population data n=104,000 Saad et al. 2019 [d]	Global RCC Study n=4288 (69.2% European cases) Laguna et al. 2014[e]
Age (years) median (range)	63.5 (29-92)	69[a]	<65 (52%)<br>>65 (48%)	62 (18-92)
Sex (M:F) (%)	65:35	63:37[b]	64:36	64:36
pT stage n (%)				
1	341 (57)	-	-	2954 (68)
2	78 (13)	-	-	553 (13)
3	172 (29)	-	-	617 (14)
4	4 (1)	-	-	32 (1)
Missing	1 (0)	-	-	195 (4)
TNM Stage n (%)				
I	341 (56)	4005 (43)[c]	68094 (65.1)	-
II	70 (12)	677 (7)	(Combined with I)	-
III	142 (23)	1560 (17)	16480 (15.8)	-
IV	55 (9)	1834 (20)	16513 (15.8)	299 (7)
Missing	0 (0)	1222 (13)	-	-
Tumour Type n (%)				
Clear cell	480 (79)	-	* 46818 (72.0)	2424 (75.9)
Papillary	60 (10)	-	8730 (13.4)	435 (13.6)
Chromophobe	46 (8)	-	4127 (6.3)	247 (7.8)
Other RCC	21 (3)	-	5354 (8.2)	87 (2.7)

[a] Shephard E et al. Clinical features of kidney cancer in primary care: a case-control study using primary care records. *Br J Gen Pract* 2013:**63**:e250-5. n=3149 UK cases
[b] Cancer Research UK. https://www.cancerresearchuk.org/health-professional/cancer-statistics-for-the-uk. 2016
[c] http://ncin.org.uk/publications/survival_by_stage. 2017.
[d] Saad et al. Trends in Renal-Cell Carcinoma Incidence and Mortality in the United States in the Last 2 Decades: A SEER-Based Study. Clin Genitourinary Cancer. 2019;**17**:46-57
* amongst 62.2% of cases with known tumour type
[e] Laguna MP, Algaba F, Cadeddu J *et al.* Current patterns of presentation and treatment of renal masses: a clinical research office of the endourological society prospective study. *J Endourol* 2014:**28**:861-70.
Prospective epidemiological, clinical and pathological data on consecutive patients with renal masses treated during a 1-year period in 98 centres worldwide

For peer review only

STROBE Statement—Checklist of items that should be included in reports of ***cohort studies***

	Item No	Recommendation	Page No
**Title and abstract**	1	(*a*) Indicate the study's design with a commonly used term in the title or the abstract<br>(*b*) Provide in the abstract an informative and balanced summary of what was done and what was found	1, 2
**Introduction**			
Background/rationale	2	Explain the scientific background and rationale for the investigation being reported	4
Objectives	3	State specific objectives, including any prespecified hypotheses	4
**Methods**			
Study design	4	Present key elements of study design early in the paper	5
Setting	5	Describe the setting, locations, and relevant dates, including periods of recruitment, exposure, follow-up, and data collection	7
Participants	6	(*a*) Give the eligibility criteria, and the sources and methods of selection of participants. Describe methods of follow-up<br>(*b*) For matched studies, give matching criteria and number of exposed and unexposed	Referenced
Variables	7	Clearly define all outcomes, exposures, predictors, potential confounders, and effect modifiers. Give diagnostic criteria, if applicable	5,6
Data sources/ measurement	8*	For each variable of interest, give sources of data and details of methods of assessment (measurement). Describe comparability of assessment methods if there is more than one group	5
Bias	9	Describe any efforts to address potential sources of bias	-
Study size	10	Explain how the study size was arrived at	7
Quantitative variables	11	Explain how quantitative variables were handled in the analyses. If applicable, describe which groupings were chosen and why	7-9
Statistical methods	12	(*a*) Describe all statistical methods, including those used to control for confounding<br>(*b*) Describe any methods used to examine subgroups and interactions<br>(*c*) Explain how missing data were addressed<br>(*d*) If applicable, explain how loss to follow-up was addressed<br>(*e*) Describe any sensitivity analyses	6 and table legends
**Results**			
Participants	13*	(a) Report numbers of individuals at each stage of study—eg numbers potentially eligible, examined for eligibility, confirmed eligible, included in the study, completing follow-up, and analysed<br>(b) Give reasons for non-participation at each stage<br>(c) Consider use of a flow diagram	7
Descriptive data	14*	(a) Give characteristics of study participants (eg demographic, clinical, social) and information on exposures and potential confounders<br>(b) Indicate number of participants with missing data for each variable of interest<br>(c) Summarise follow-up time (eg, average and total amount)	Tables P7
Outcome data	15*	Report numbers of outcome events or summary measures over time	Figures

For peer review only

Main results	16	(*a*) Give unadjusted estimates and, if applicable, confounder-adjusted estimates and their precision (eg, 95% confidence interval). Make clear which confounders were adjusted for and why they were included<br>(*b*) Report category boundaries when continuous variables were categorized<br>(*c*) If relevant, consider translating estimates of relative risk into absolute risk for a meaningful time period	NA
Other analyses	17	Report other analyses done—eg analyses of subgroups and interactions, and sensitivity analyses	-
**Discussion**			
Key results	18	Summarise key results with reference to study objectives	10-14
Limitations	19	Discuss limitations of the study, taking into account sources of potential bias or imprecision. Discuss both direction and magnitude of any potential bias	3<br>10-14
Interpretation	20	Give a cautious overall interpretation of results considering objectives, limitations, multiplicity of analyses, results from similar studies, and other relevant evidence	10-14
Generalisability	21	Discuss the generalisability (external validity) of the study results	10-14
**Other information**			
Funding	22	Give the source of funding and the role of the funders for the present study and, if applicable, for the original study on which the present article is based	14

*Give information separately for exposed and unexposed groups.

Note: An Explanation and Elaboration article discusses each checklist item and gives methodological background and published examples of transparent reporting. The STROBE checklist is best used in conjunction with this article (freely available on the Web sites of PLoS Medicine at http://www.plosmedicine.org/, Annals of Internal Medicine at http://www.annals.org/, and Epidemiology at http://www.epidem.com/). Information on the STROBE Initiative is available at http://www.strobe-statement.org.

For peer review only

For peer review only - http://bmjopen.bmj.com/site/about/guidelines.xhtml
